# Single-cell analyses implicate ascites in remodeling the ecosystems of primary and metastatic tumors in ovarian cancer

**DOI:** 10.1038/s43018-023-00599-8

**Published:** 2023-07-24

**Authors:** Xiaocui Zheng, Xinjing Wang, Xi Cheng, Zhaoyuan Liu, Yujia Yin, Xiaoduan Li, Zhihao Huang, Ziliang Wang, Wei Guo, Florent Ginhoux, Ziyi Li, Zemin Zhang, Xipeng Wang

**Affiliations:** 1 Department of Obstetrics and Gynecology, Xinhua Hospital, Shanghai Jiaotong University School of Medicine, Shanghai, China; 2 Department of Gynecological Oncology, Fudan University Shanghai Cancer Center, Shanghai, China; 3 Department of Immunology and Microbiology, Shanghai Institute of Immunology, Shanghai Jiao Tong University School of Medicine, Shanghai, China; 4 Analytical Biosciences Limited, Beijing, China; 5 Singapore Immunology Network (SIgN), Agency for Science, Technology and Research (A∗STAR), Singapore, Singapore; 6 Translational Immunology Institute, SingHealth Duke-NUS Academic Medical Centre, Singapore, Singapore; 7 Gustave Roussy Cancer Campus, Villejuif, France; 8 BIOPIC, and School of Life Sciences, Peking University, Beijing, China

**Keywords:** Ovarian cancer, Tumour heterogeneity, Tumour immunology, Cancer

## Abstract

Ovarian cancer (OC) is an aggressive gynecological tumor usually diagnosed with widespread metastases and ascites. Here, we depicted a single-cell landscape of the OC ecosystem with five tumor-relevant sites, including omentum metastasis and malignant ascites. Our data reveal the potential roles of ascites-enriched memory T cells as a pool for tumor-infiltrating exhausted CD8^+^ T cells and T helper 1-like cells. Moreover, tumor-enriched macrophages exhibited a preference for monocyte-derived ontogeny, whereas macrophages in ascites were more of embryonic origin. Furthermore, we characterized MAIT and dendritic cells in malignant ascites, as well as two endothelial subsets in primary tumors as predictive biomarkers for platinum-based chemotherapy response. Taken together, our study provides a global view of the female malignant ascites ecosystem and offers valuable insights for its connection with tumor tissues and paves the way for potential markers of efficacy evaluation and therapy resistance in OC.

## Abstract (web summary)

Wang and colleagues perform single-cell profiling of human ovarian cancer samples from five anatomic sites, revealing dynamics of the immune microenvironment in malignant ascites and cell subtypes that may play a role in chemotherapy response.

## Main

As a heterogeneous disease, ovarian cancer (OC) is the most lethal gynecological malignancy, which accounts for 5% of cancer deaths in females^1^. OC is a heterogeneous disease consisting of malignancies with different histological subtypes, molecular biology and microenvironment features, which affect its treatment response and clinical outcomes^2^. Among all OC types, high-grade serous OC (HGSOC) is the most common histological subtype accounting for more than 70% of patients with OC^3^. Once diagnosed, over 75% of patients with HGSOC present an advanced disease with widespread metastasis and ascites^4,5^. As reported, a predilection of metastasis to omentum in OC is consistently identified owing to the fatty structure of omentum and peritoneal circulation^6^. Although treatments with chemotherapy plus bevacizumab prolong the 5-year survival, the overall benefits are still limited. Additionally, immunotherapies such as immune-checkpoint inhibitors only showed an objective response rate of 10% in clinical trials^7^ and OC subtypes often exhibited diverse responses to immunotherapy owing to the different proportion and quality of tumor-infiltrating lymphocytes (TILs)^8,9^. Therefore, it is essential to characterize the tumor microenvironment (TME) of OC, which harbors diverse cellular components playing important roles in disease progression and therapy response.

Single-cell mRNA sequencing (scRNA-seq) is a powerful tool to characterize the cellular features and dynamic relationships of different cell populations in multiple malignancies^10–12^. For instance, a previous single-cell atlas of primary ovarian tumor has revealed a *GZMK*^+^ CD8^+^ effector memory (T_EM_) T cell subset as pre-dysfunctional effector memory cells^13^. Moreover, another OC study defined a population of stem cell-like tissue-resident memory T cells with a maximal expression level of *GZMK*, which would develop into exhausted T (T_EX_) cells^14^; however, where these memory T cells originate from is still unknown due to the limited sampling tissues in previous studies. Besides primary tumors, omentum metastases and malignant ascites are equally important in OC studies. For example, interleukin (IL)-6 secreted from cancer-associated fibroblasts in the ascites ecosystem could stimulate JAK–STAT signaling in malignant cells, leading to a poor prognosis and resistance to chemotherapies^15^. But previous single-cell analysis of OC ascites focused largely on malignant cells and other CD45^−^ cells^15^ and little is known about the immune milieu in the OC ascites and how malignant ascites influence the immune status of OC. Thus, a high-resolution cellular landscape involving multiple-site tissues is needed to characterize the comprehensive TME of different OC sites, especially omentum metastasis and ascites.

Here, we delineated a comprehensive landscape of OC TME via scRNA-seq by comparing the unique cellular compositions of five tumor-related sites, including primary ovarian tumor (Pri.OT), omentum metastasis (Met.Ome), ascites, pelvic lymph node (PLN) and peripheral blood (PB). Through T cell receptor (TCR)-based lineage tracing and trajectory inference, we unveiled potential dynamic characteristics of T cells from ascites to tumor tissues. We characterized the functional states and ontogeny of macrophages in ascites and tumor tissues and also highlighted *DES*^+^ mesothelial cells as important immunoregulators reprogramming OC ascites. Additionally, we revealed the associations between distinct cellular compositions and the clinical responses to platinum-based chemotherapy, which might serve as indicators of treatment effectiveness. Taken together, our findings provide insights into the functions of malignant ascites and would provide an important resource to guide the development of additional therapeutic strategies.

## Results

### High-resolution landscape of OC by multisite scRNA-seq

**Fig. 1:**
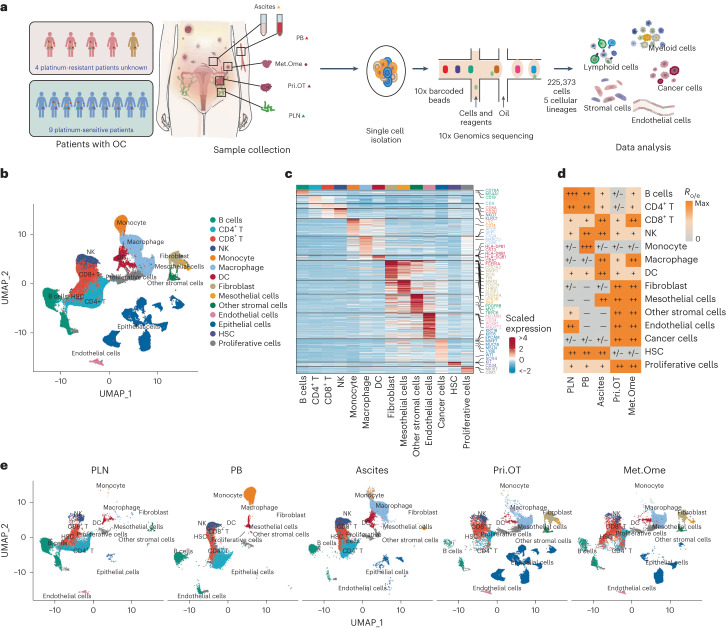
Landscape of advanced ovarian cancer via scRNA-seq of five sites. **a**, Overall study design with flowchart of sample collection and single-cell analysis of OC by 10x Genomics sequencing. *n* = 14 patients with OC who were responsive or nonresponsive to platinum-based chemotherapy were recruited to our study. In total, *n* = 39 samples, including *n* = 6 PB, *n* = 5 PLN, *n* = 13 Pri.OT, *n* = 5 matched Met.Ome and *n* = 10 ascites samples were analyzed. Each dot corresponds to one sample, colored by sample types. Red triangle, orange triangle, dark red circle, dark red triangle, green triangle represent blood, ascites, primary tumor, omentum metastases and pelvic lymph node, respectively. **b**, Uniform Manifold Approximation and Projection (UMAP) plot showing 14 clusters of *n* = 10 patients with HGSOC identified by integrated analysis. Each dot corresponds to a single cell, colored by clusters. NK, natural killer; HSC, hematopoietic stem cell. **c**, Heat map depicting expression levels of selected highly expressed genes (including marker genes) across major clusters of HGSOC. Rows represent genes and columns represent clusters. **d**, Tissue preference of each major cluster in HGSOC estimated by *R*_o/e_. **e**, UMAP plots showing the distinct cell composition of five different sample sites in patients with HGSOC. For **b**–**e**, a total of *n* = 31 HGSOC samples, including *n* = 5 PB, *n* = 4 PLN, *n* = 10 Pri.OT, *n* = 4 Met.Ome and *n* = 8 ascites samples were analyzed. Source data

**Extended Data Fig. 1:**
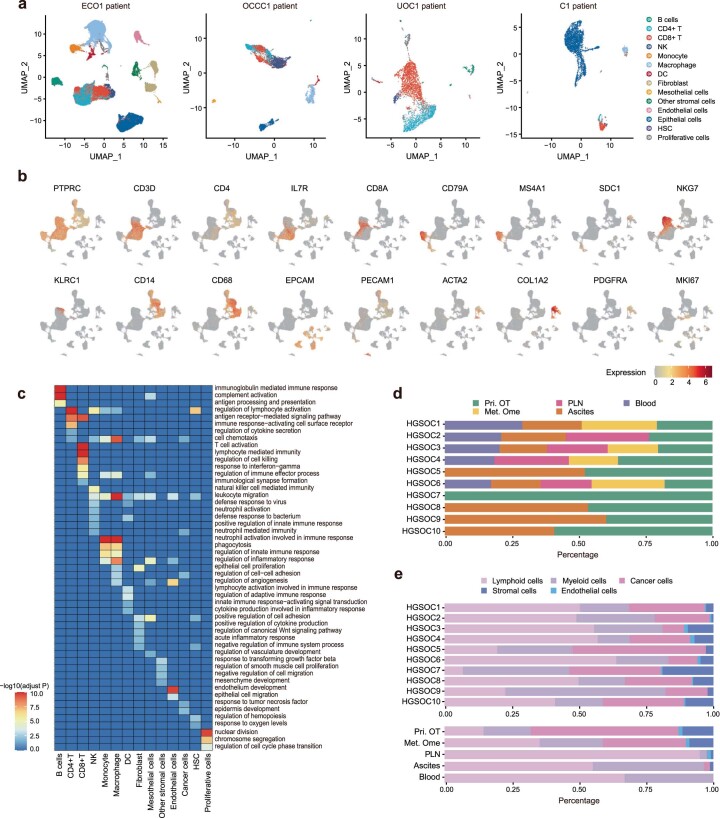
Basic information of 14 major clusters in OC. **a**, UMAP plots showing 14 clusters of other OC subtypes identified by integrated analysis. Data were summarized from n = 4 patients of other ovarian tumor types. OCCC1: Ovarian clear cell carcinomas, ECO1: Endometrioid carcinoma of the ovary, UOC1: Undifferentiated ovarian cancer, C1: ovarian carcinosarcoma. Each dot corresponds to a single cell, colored by clusters. **b**, UMAP plots showing expression levels of highly expressed genes (including cluster-specific marker genes) in 14 major cell clusters using data of HGSOC patients. **c**, Heat map depicting selected activated pathways across major clusters using data of HGSOC patients. Rows represent pathways and columns represent clusters. Pathways, GO, adjusted P value by Benjamini-Hochberg (BH) procedure < 0.05. **d**, Tissue distribution of detected cells in each n = 10 HGSOC patient, colored by tissues. **e**, Patient distribution (upper) and tissue distribution (lower) of each major cluster detected in n = 10 HGSOC patients, colored by clusters. For **b-e**, totally n = 31 HGSOC samples including n = 5 peripheral blood, n = 4 pelvic lymph node (PLN), n = 10 primary ovarian tumor (Pri.OT), n = 4 matched omentum metastatic tumor (Met.Ome), and n = 8 ascites samples were used for analysis. Source data

To elucidate the complexity of cellular compositions in ovarian cancer, we utilized scRNA-seq to analyze unsorted cells from PB, PLN, Pri.OT, matched Met.Ome and malignant ascites of 14 patients with advanced OC (Fig. 1a and Supplementary Table 1). These patients exhibited five distinct histological subtypes and varying responses to platinum-based chemotherapy. In total, we cataloged 223,363 high-quality single cells into five major cell lineages annotated by canonical marker expression (Fig. 1b,c, Extended Data Fig. 1a–c and Supplementary Table 2).

We first quantified relative tissue enrichment of major cell clusters by calculating the ratio of observed to expected cell numbers (*R*_o/e_) using data of patients with HGSOC (Fig. 1d,e, Extended Data Fig. 1d and Supplementary Table 3). As expected, B cells and CD4^+^ T cells dominated the PLNs, whereas lymphocytes and monocytes constituted the main cellular components of PB samples. Of note, we identified all five major cell lineages in both Pri.OT and Met.Ome and the enrichment pattern of most cell types showed no significant differences between these two sites, suggesting a similar complex TME necessary to the development of both primary and metastatic tumor cells (Fig. 1e and Extended Data Fig. 1e). Ascites, frequently found in patients with advanced OC and associated with chemotherapy response^5^, harbored a large number of immune cells and stromal cells. Among them, CD8^+^ T cells, macrophages and dendritic cells (DCs) were major constituents of ascites with high cell proportions, indicating an inflammatory microenvironment. Mesothelial cells, recently reported to be tightly associated with metastasis of OC^16^, were also preferentially found in malignant ascites (Fig. 1d,e).

**Extended Data Fig. 2:**
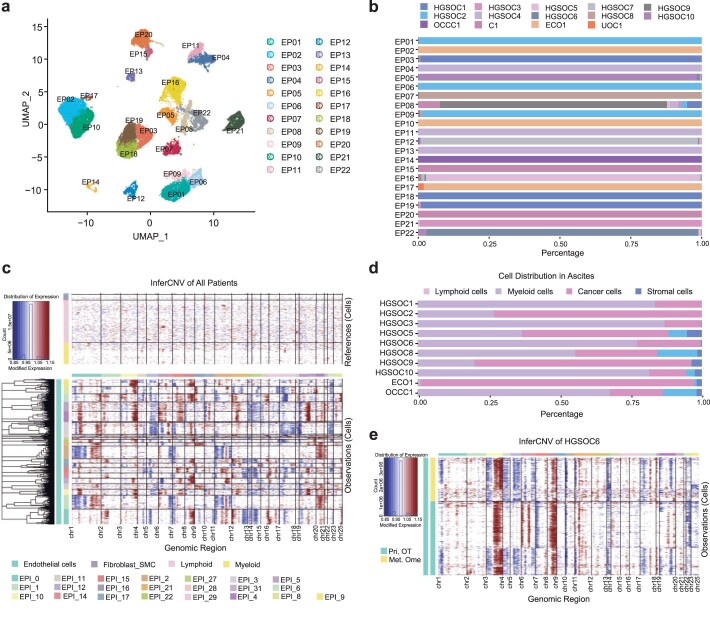
Clustering and characterization of malignant cells in all OC patients. **a**, UMAP plot showing 22 epithelial clusters identified by integrated analysis. Each dot corresponds to a single cell, colored by clusters. **b**, Patient distribution of each epithelial cell cluster, colored by patients. **c**, Heat map showing large-scale inferred copy number variations (inferCNV) for individual cells in 22 epithelial clusters (row) based on the average expression of 100 genes surrounding each chromosomal position (column), compared to non-cancer clusters as a reference. CNVs in red indicates amplifications, and blue indicates deletions. **d**, Proportion of each major cluster in ascites of each patient, colored by major clusters. Malignant epithelial cells were confirmed by inferCNV, referred by Extended Data Fig. 2c. **e**, Heat map showing inferCNV for individual epithelial cells from patient HGSOC6. CNVs in red indicates amplifications, and blue indicates deletions. For **a-d**, totally n = 39 samples from patients with all ovarian tumor types were used for analysis, including n = 6 peripheral blood, n = 5 pelvic lymph node (PLN), n = 13 primary ovarian tumor (Pri.OT), n = 5 matched omentum metastatic tumor (Met.Ome), and n = 10 ascites samples.

Unlike nonmalignant cells, tumor cells as defined by inferred copy number variations (inferCNV), exhibited a strong interpatient heterogeneity (Extended Data Fig. 2a–c). Notably, tumor cells were identified in all ascites samples, with an averaged proportion of 2.7% (1,444 of 53,499) (Extended Data Fig. 2d). Our observation was consistent with the notion that OC tumor cells prefer to ‘seed’ to the peritoneal cavity rather than spreading via vasculature, which highlights the tight association between ascites and intraperitoneal spread of OC^17^. Further, inferCNV analyses showed that the subclones of tumor cells found within Met.Ome were also detectable in that of Pri.OT (Extended Data Fig. 2e), indicating these subclones as tumorigenic populations of peritoneal metastasis.

### Dynamic relationships of T cells in OC

**Fig. 2:**
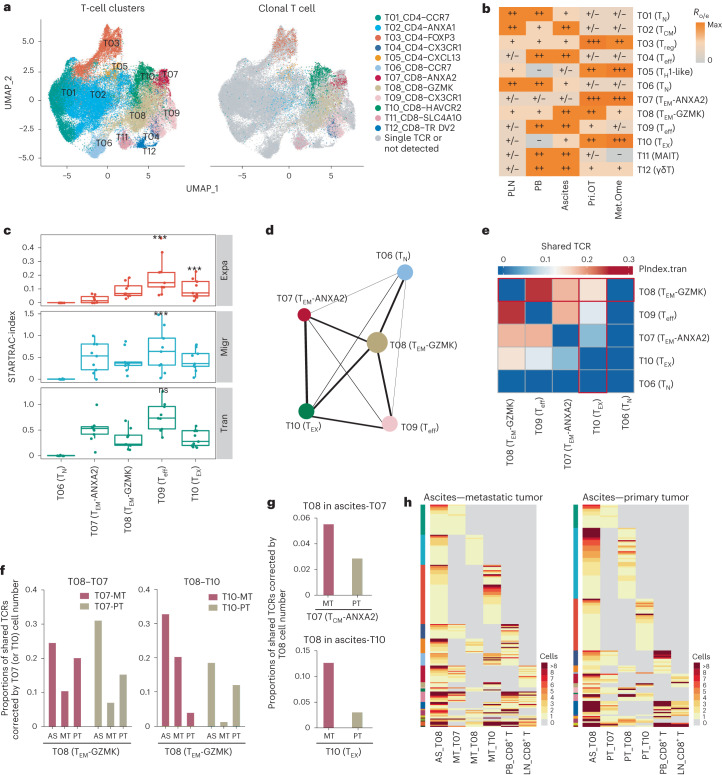
Characterization of T cell clusters and dynamics of CD8^+^ T cells in HGSOC. **a**, UMAP plots showing 12 clusters of T cells and clonal T cells within each cluster, colored by clusters. **b**, Tissue preference of each T cell cluster estimated by *R*_o/e_. **c**, Clonal expansion, migration and transition potential of CD8^+^ T cells quantified by STARTRAC indices. Indices were quantified for *n* = 9 patients with more than two matched samples. Center line indicates the median value, lower and upper hinges represent the 25th and 75th percentiles, respectively and whiskers denote 1.5 × interquartile range. *P < 0.05, **P < 0.01, ***P < 0.001; permutation test (exact *P* values are provided in source data). **d**, PAGA analysis of CD8^+^ T cells. Each dot represents a T cell cluster. **e**, Heat map showing the developmental transition potential between CD8^+^ T cells quantified by pairwise STARTRAC-tran indices. The horizontal red box represents the transition between *GZMK*^+^ T_EM_ and other CD8^+^ T cells and the vertical red box refers to the transition between other CD8^+^ T cells and T_EX_ cells. **f**, Bar plots showing proportions of shared TCRs between *GZMK*^+^ T_EM_ (T08) and *ANXA2*^+^ T_EM_ (T07) (left) or T_EX_ (T10) (right) corrected by cell numbers of *ANXA2*^+^ T_EM_ (T07) or T_EX_ (T10) in sampled tissues, respectively. **g**, Bar plots showing proportions of shared TCRs between *GZMK*^+^ T_EM_ (T08) and *ANXA2*^+^ T_EM_ (T07) (top) or T_EX_ (T10) (bottom) corrected by cell numbers of *GZMK*^+^ T_EM_ (T08) in ascites. **h**, The distribution of clonal clonotypes in indicated CD8^+^ subsets derived from ascites and two tumor sites. For **a**,**b**,**d**, data were summarized from all *n* = 31 HGSOC samples. For **c**,**e**–**h**, all *n* = 30 HGSOC samples except for the primary tumor sample of HGSOC7 were analyzed. AS, ascites; PT, primary ovarian tumor; MT, omentum metastatic tumor. Source data

**Extended Data Fig. 3:**
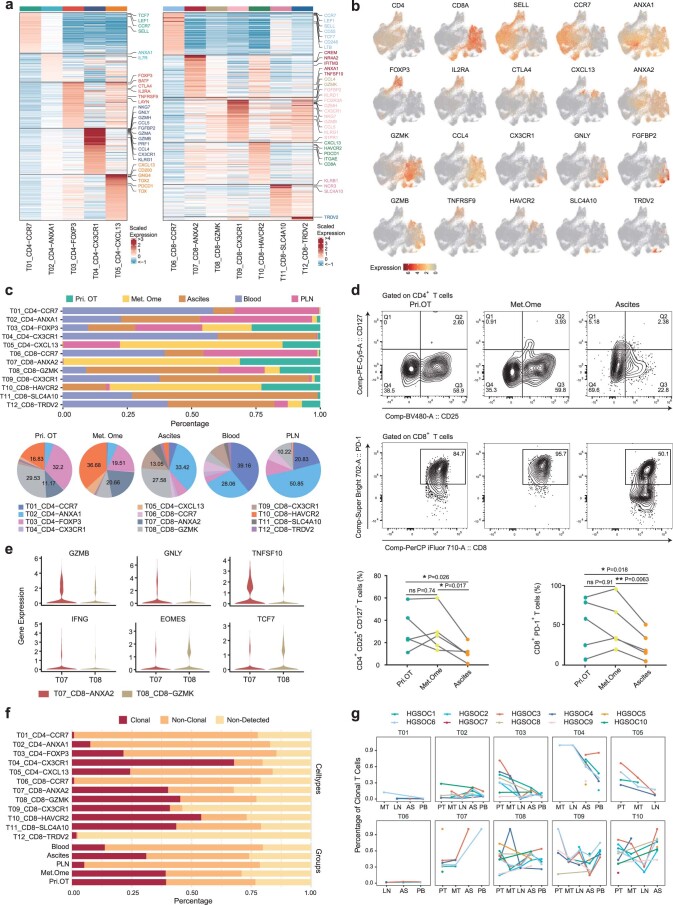
Gene expression, tissue distribution and clonal types of T-cell clusters. **a**, Heatmap showing selected highly expressed genes of 12 T cell clusters. Rows represent genes and columns represent clusters. **b**, UMAP plots showing selected marker genes of 12 T cell clusters. Each dot corresponds to a single cell. **c**, Tissue distribution of each T cell cluster, colored by different tissues (upper) and clusters (lower). **d**, Representative flow-cytometric plots, and summary data of frequency of CD25^+^ CD127^+^ CD4^+^ T cells (Treg) and PD-1^+^ CD8^+^ T cells in the microenvironment of n = 5 primary tumors, n = 5 matched omentum metastasis and n = 5 ascites samples from 5 HGSOC patients. P values were determined by paired one-sided t-test. *P < 0.05, **P < 0.01, ***P < 0.001. **e**, Violin plots showing the differentially expressed genes of two CD8^+^ Tem clusters with distinct tissue preference. CD8-ANXA2 was enriched in tumor sites and CD8-GZMK was enriched in ascites. **f**, The proportion of each T cell clonotype in each T cell cluster (upper) and tissue distribution of each clonotype (lower), colored by clonotypes. **g**, The proportion of clonal T cells in each cluster and different tissues, each color represents a patient diagnosed with HGSOC. AS: ascites, PT: primary ovarian tumor, MT: metastatic ovarian tumor, PB: peripheral blood, LN: lymph node. For **a-c** and **e-g**, data were summarized from all n = 31 HGSOC samples. Source data

Given that HGSOC is the most common OC subtype, we focused on HGSOC in the subsequent analyses of specific cellular compartments in the TME. We first focused on the intrinsic properties and potential functions of T cell populations in OC. By unsupervised clustering, we identified five CD4^+^ clusters, five CD8^+^ clusters and two unconventional clusters (Fig. 2a, Extended Data Fig. 3a,b and Supplementary Table 4). The conventional T cell clusters were further split into naive (T_N_), central memory (T_CM_), effector memory (T_EM_), effector (T_eff_), regulatory (T_reg_), T helper 1 (T_H_1)-like^18^ and exhausted^19^ (T_EX_) T cell clusters, which showed different tissue preference patterns (Fig. 2a,b, Extended Data Fig. 3c and Supplementary Table 5). T_N_ cells (T01 and T06) were enriched in PB and PLN, maintaining a quiescent state. Consistent with previous studies^20^, the majority of immunosuppressive *FOXP3*^+^ T_reg_ (T03) cells and the *HAVCR2*^*+*^ exhausted CD8^+^ cells (T10), were predominantly enriched in both two tumor sites. The analyses by flow cytometry also suggested a higher proportion of T_reg_ and PD-1^+^ T cells in tumor sites than in ascites (Extended Data Fig. 3d), further proving a more immunosuppressive status in tumor tissues compared to malignant ascites. Additionally, *CXCL13*^*+*^ T_H_1-like cells (T05) were also enriched in tumor sites, whereas CD4^+^*ANXA1*^+^ T_CM_ (T02) and *CX3CR1*^+^ T_eff_ cells (T04 and T09) were mainly detected in blood and ascites. Specifically, we identified two CD8^+^ T_EM_ clusters occupying a large proportion of CD8^+^ T cells, with T07 *ANXA2*^+^ T_EM_ enriched in tumor sites and T08 *GZMK*^+^ T_EM_ enriched in ascites (Fig. 2b). Based on limited differential expressed genes, we observed that tumor-enriched *ANXA2*^+^ T_EM_ cells expressed increased levels of genes encoding effector molecules (such as *GNLY*, *GZMB* and *TNFSF10*)^12^ (Extended Data Fig. 3e), indicating the intrinsic antitumor effector potential of T_EM_ cells inside tumors. By contrast, ascites-enriched *GZMK*^+^ T_EM_ cells exhibited higher expressions of *EOMES* and *TCF7* (ref. ^21^) (Extended Data Fig. 3e), which are the key transcription factor genes in progenitor T_EX_ cells, suggesting that *GZMK*^+^ T_EM_ cells were more likely to transit into T_EX_ cells.

**Extended Data Fig. 4:**
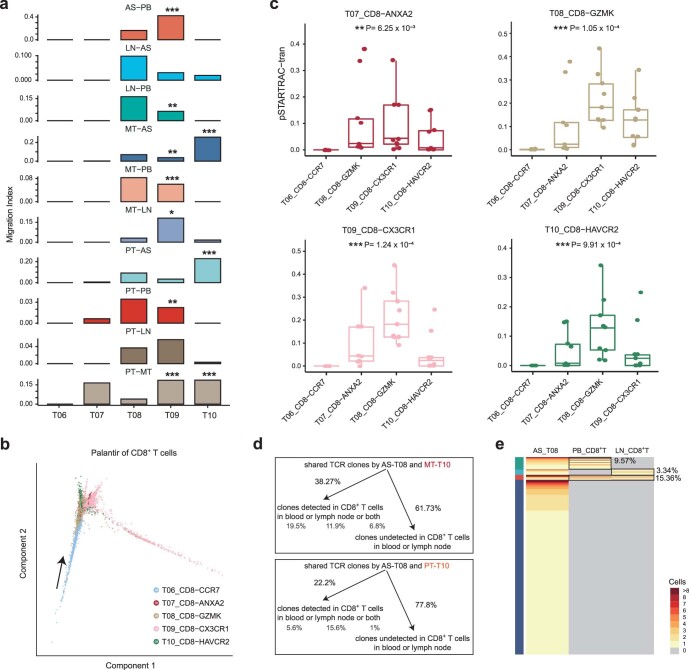
CD8^+^ T cell analyses based on integrated expression and TCR clonality. **a**, Migration potential of CD8^+^ T cell clusters across different tissues quantified by pairwise STARTRAC-migr indices. Data were presented as mean value. *P < 0.05, **P < 0.01, ***P < 0.001, permutation test (exact P values were provided in source data). **b**, UMAP plot showing the developmental trajectories of CD8^+^ T cells by Palantir analysis. Each dot corresponds to a single cell, each color represents a T cell cluster. The arrow represents the direction of cell differentiation with naïve T cells as initial cluster. Data were summarized from all n = 31 HGSOC samples. **c**, pSTARTRAC-tran indices of CD8-ANXA2, CD8-CX3CR1, CD8-HAVCR2 and CD8-GZMK cells for each n = 9 HGSOC patient with matched tissue samples, depicted by dots. Center line indicates the median value, lower and upper hinges represent the 25th and 75th percentiles, respectively, and whiskers denote 1.5× interquartile range. Ns non-significant, *P < 0.05, **P < 0.01, ***P < 0.001, Kruskal–Wallis test. **d**, Diagram showing the proportions of TCR clones shared by ascites Tem (T08) and Tex cells (T10) from metastatic tumor (MT) or primary tumor (PT) which could be detected in blood or lymph node-derived T cells, related to Fig. 2h. **e**, Heatmap showing the shared TCRs between ascites-derived Tem (T08), and blood or lymph node-derived CD8^+^ T cells. For **a** and **c-e**, all n = 30 HGSOC samples except for the primary tumor sample of HGSOC7 were used for analysis. AS: ascites, PT: primary ovarian tumor, MT: omentum metastatic tumor, PB: peripheral blood, LN: pelvic lymph node. Source data

Combined with TCR-seq and single-cell transcriptomics, we captured at least one pair of full-length productive α- and β-chains in 54,061 T cells, of which 21.12% (11,415 cells) harbored repeated TCRs of 2,386 clonotypes (Fig. 2a and Extended Data Fig. 3f,g). We then quantitatively evaluated the T cell dynamics using the previously developed STARTRAC indices upon TCR tracking^18^ (Methods). T cells carrying repetitive TCRs are defined as clonal cells. The presence of clonal cells across several different tissue sites within the same cluster implies the tissue migration (STARTRAC-migr) of indicated T cell subtypes. And clonal cells found within a T cell cluster were quantified with STARTRAC-expa index, whereas clonal cells between two different T cell subtypes referred to cell state transition (STARTRAC-tran). Among all CD8^+^ T cells, T_eff_ cells showed the highest clonal expansion, migration and transition index (Fig. 2c), as expected. Additionally, expa index pointed out that clonal expansion might be a possible explanation for the T_EX_ enrichment in tumor sites (Fig. 2c), consistent with previous findings^22^. Notably, we observed strong TCR sharing of T_EX_ cells among two tumor sites and ascites (Met.Ome-AS, Pri.OT-AS and Pri.OT-Met.Ome) (Extended Data Fig. 4a). Considering that exhausted T cells had poor migration capability^18^, this was seemingly logical as these T_EX_ cells would recognize the same tumor-derived neoantigens in different tissues.

To decipher the potential developmental trajectories of T cells, we performed PAGA^23^ and Palantir^24^ analysis, excluding two unconventional clusters due to their distinct TCR characteristics. We noticed that ascites-enriched *GZMK*^+^ T_EM_ (T08) was located centrally bridging T_N_ (T06), T_EX_ (T09) and T_eff_ (T10) cells (Fig. 2d and Extended Data Fig. 4b), indicating their intermediate states. In addition, STARTRAC pairwise transition analysis based on TCR sharing also showed that *GZMK*^+^ T_EM_ exhibited a high ability of transition to T_eff_, *ANXA2*^+^ T_EM_ and T_EX_ cells (Fig. 2e and Extended Data Fig. 4c), further supporting our inferred trajectory analyses. As reported, CD8^+^ *GZMK*^+^ T cells were defined as ‘pre-exhausted’ cells within tumors, which were accumulated by local expansion and replenishment and could further transit to terminal exhausted T cells^11,25^. Likewise, compared to other T cells, T08 *GZMK*^+^ T_EM_ in our study also harbored a higher ability to transit into T_EX_ cells (Fig. 2e and Extended Data Fig. 4c), suggesting transition from *GZMK*^+^ T_EM_ as an important source of T_EX_ cells. Given that *GZMK*^+^ T_EM_ cells were mostly enriched in ascites, their transitions to tumor-enriched clusters (T_EX_ and *ANXA2*^+^ T_EM_) might happen together with cross-tissue migration. Thus, we further checked TCR sharing between *GZMK*^+^ T_EM_ and T_EX_/*ANXA2*^+^ T_EM_ across different tissues and found that T_EX_ and *ANXA2*^+^ T_EM_ cells in tumor sites shared more TCR clones with ascites-derived *GZMK*^+^ T_EM_ cells than tumor-derived *GZMK*^+^ T_EM_ cells (Fig. 2f). The results indicated that ascites-derived *GZMK*^+^ T_EM_ cells might serve as an important source of T cells infiltrating into tumor sites and further transit into T_EX_ or *ANXA2*^+^ T_EM_. Furthermore, *GZMK*^+^ T_EM_ in ascites shared more TCR clones with T_EX_ or *ANXA2*^+^ T_EM_ cells in Met.Ome than in Pri.OT (Fig. 2g), reflecting a preference of ascites-derived *GZMK*^+^ T_EM_ infiltrating into Met.Ome. Then, we checked the TCRs shared among ascites-derived T_EM_ (T08) and tumor-derived T07, T08 and T_EX_ (T10) to confirm the connections between ascites T_EM_ and tumor T_EX_ cells. Of note, tumor T_EX_ (T10) clones linked to ascites-derived *GZMK*^+^ T_EM_ showed mutually exclusive patterns with tumor T10 clones linked to T07 and T08 clusters in tumors (Fig. 2h). Considering the hard-to-reverse nature of exhaustion and the lack of mobility of T_EX_ cells, these results further support the notion that T_EX_ cells in tumor may be derived from *GZMK*^+^ T_EM_ in ascites, in a process including cross-tissue migration and state transition. Moreover, we checked whether the TCR clones shared by ascites T_EM_ (T08) and tumor T_EX_ (T10) also existed in blood or lymph nodes. We found that the majority of TCR clones shared by ascites T_EM_ (T08) and T_EX_ cells (T10) from primary tumor (61.73%) or metastatic tumor (77.8%) could not be detected in blood or lymph node-derived T cells (Fig. 2h and Extended Data Fig. 4d), further supporting the idea that ascites T_EM_ cells could be an important direct source for TILs. To find the clues about where these TCR clones that are undetected in blood/lymph nodes might come from, we examined the origins of all ascites-enriched T_EM_ cells (T08). We found that the TCRs in 15.36% ascites T_EM_ (T08) cells could be detected in both blood and lymph nodes, whereas 9.57% and 3.34% ascites clonal T_EM_ shared TCRs only with blood T cells or lymph node T cells, respectively (Extended Data Fig. 4e). Taken together, these findings provide insights into the cycle of CD8^+^ T cells in OC and suggest that ascites-derived *GZMK*^+^ T_EM_ cells might serve as a direct source of tumor-infiltrating T_EX_ cells.

**Fig. 3:**
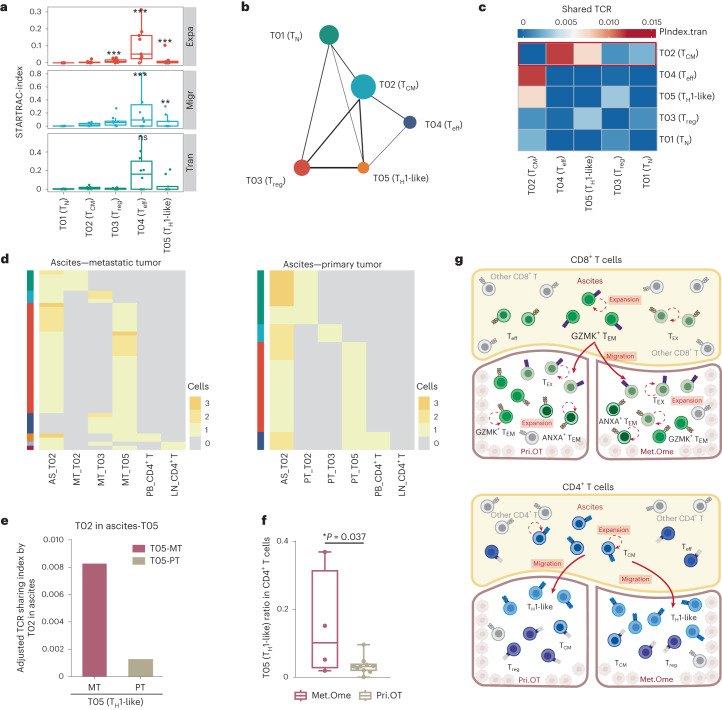
Characterization and dynamics of CD4^+^ T cells in HGSOC. **a**, Clonal expansion, migration and transition potential of CD4^+^ T cells quantified by STARTRAC indices. Indices were quantified for each *n* = 9 patient with more than two matched samples. Center line indicates the median value, lower and upper hinges represent the 25th and 75th percentiles, respectively and whiskers denote 1.5 × interquartile range. *P < 0.05, **P < 0.01, ***P < 0.001; permutation test (exact *P* values are provided in source data). **b**, PAGA analysis of CD4^+^ T cells. Each dot represents a T cell cluster. In total *n* = 31 HGSOC samples were used for analysis. **c**, Heat map showing the developmental transition potential between CD4^+^ T cells quantified by pairwise STARTRAC-tran indices. The red box represents the transition between T_CM_ and other CD4^+^ T cells. **d**, The distribution of clonal clonotypes in indicated CD4^+^ subsets derived from ascites and two tumor sites. **e**, Bar plots showing proportions of shared TCRs between T_CM_ (T02) and T_H_1-like cells (T05) corrected by cell numbers of T_CM_ (T02) in ascites, related to Extended Data Fig. 5e. **f**, Frequency of T_H_1-like cells as a proportion of all CD4^+^ T cells in *n* = 4 Met.Ome and *n* = 10 Pri.OT samples from ten patients with HGSOC. Center line indicates the median value, lower and upper hinges represent the 25th and 75th percentiles, respectively and whiskers indicates min to max. *P < 0.05, **P < 0.01, ***P < 0.001; unpaired two-sided *t*-test. **g**, Sketch map showing the dynamics of CD8^+^ T cells (top) and CD4^**+**^ T cells (bottom) between ascites and two tumor sites. For **a**,**c**–**e**, data were summarized from all *n* = 30 HGSOC samples except for the primary tumor sample of HGSOC7. Source data

**Extended Data Fig. 5:**
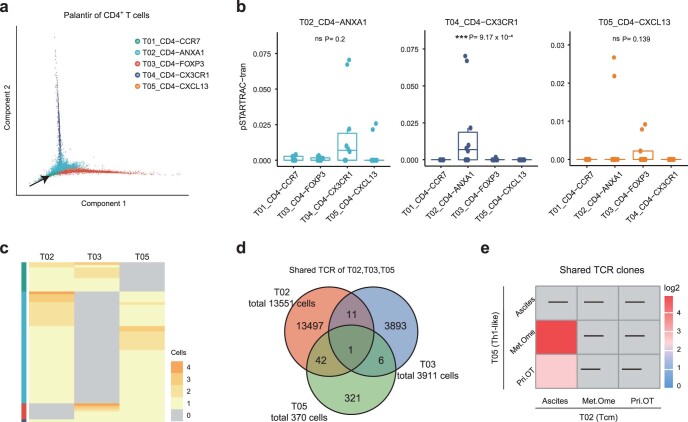
CD4^+^ T cell analyses based on integrated expression and TCR clonality. **a**, UMAP plot showing the developmental trajectories of CD4^+^ T cells by Palantir analysis. Each dot corresponds to a single cell, each color represents a T cell cluster. The arrow represents the direction of cell differentiation with naïve T cells as initial cluster. Data were summarized from all n = 31 HGSOC samples. **b**, pSTARTRAC-tran indices of CD4-ANXA1, CD4-CX3CR1 and CD4-CXCL13 cells for each n = 9 HGSOC patient with matched tissue samples, depicted by dots. Center line indicates the median value, lower and upper hinges represent the 25th and 75th percentiles, respectively, and whiskers denote 1.5× interquartile range. Ns non-significant, *P < 0.05, **P < 0.01, ***P < 0.001, Kruskal–Wallis test. **c**, The distribution of clonal clonotypes in indicated CD4^+^ T subsets (T02, T03 and T05), related to Fig. 3c. **d**, Venn diagram showing the quantification of shared TCR between indicated CD4^+^ T subsets referred as in Extended Data Fig. 5c. **e**, Heatmap showing TCR sharing patterns between Th1-like (T05) and Tcm (T02) in different tissues, including ascites, Pri.OT (primary tumor) and Met.Ome (omentum metastasis). For **b-e**, all n = 30 HGSOC samples except for the primary tumor sample of HGSOC7 were used for analysis. Source data

Similar analyses were also performed on CD4^+^ T cells to quantify their tissue distributions and TCR sharing. In contrast to CD8^+^ T cells, CD4^+^ T cells showed an overall lower clonal expansion. Among these clusters, CD4^+^ T_eff_ cells exhibited the highest clonal expansion, migration and transition indexes (Fig. 3a), similar to the observations in CD8^+^ T cells. The inferred developmental trajectories also exhibited a similar branched structure. T_N_ (T01), T_H_1-like (T05) and T_reg_ (T03) cells were positioned at three different branches whereas T_CM_ (T02) cells were located in the middle (Fig. 3b and Extended Data Fig. 5a). In addition, pairwise transition analysis based on TCR sharing (Fig. 3c and Extended Data Fig. 5b) and the shared TCR pattern among T02, T03 and T05 (Extended Data Fig. 5c,d) also revealed that T_CM_ cells were associated with T_eff_ and T_H_1-like cells, suggesting T_CM_ as potential precursors of *CXCL13*^+^ T_H_1-like cells. Given that T_CM_ cells were enriched in ascites, whereas T_H_1-like cells were enriched in tumors (Fig. 2b), their transition was accompanied by the ascites to tumors cross-tissue migration of CD4^+^ memory T cells. Then, we noticed that the TCR clones shared by tumor T_H_1-like cells and T_CM_ in ascites were almost undetected in any other T cells from tumor, blood and lymph nodes (Fig. 3d and Extended Data Fig. 5e), implying that ascites-derived T_CM_ cells might be a direct source of T_H_1-like cells in tumors. Additionally, we observed more shared TCR clones between T_CM_ in ascites and T_H_1-like cells in Met.Ome compared to that in Pri.OT (Fig. 3e and Extended Data Fig. 5e), suggesting that ascites-derived T_CM_ cells were more likely to infiltrate into Met.Ome. Such a tissue preference of T_CM_ cell infiltration could be a potential explanation for the relative enrichment of T_H_1-like cells in Met.Ome than Pri.OT (Fig. 3f).

Collectively, through integrated analysis of single-cell transcriptome and TCR data, we identified multiple T cell populations with distinct distribution patterns and revealed unique dynamics of T cells from ascites to tumor sites in OC. We found that ascites-enriched memory T cells (CD8^+^ *GZMK*^+^ T_EM_ and CD4^+^ T_CM_) could be a potential important pool for TILs, including CD8^+^ T_EX_ and CD4^+^ T_H_1-like cells, especially for Met.Ome (Fig. 3g). These results implicate a potential role of ascites in shaping the TME of OC during T cell infiltration.

### DC subsets show tissue-specific patterns

**Fig. 4:**
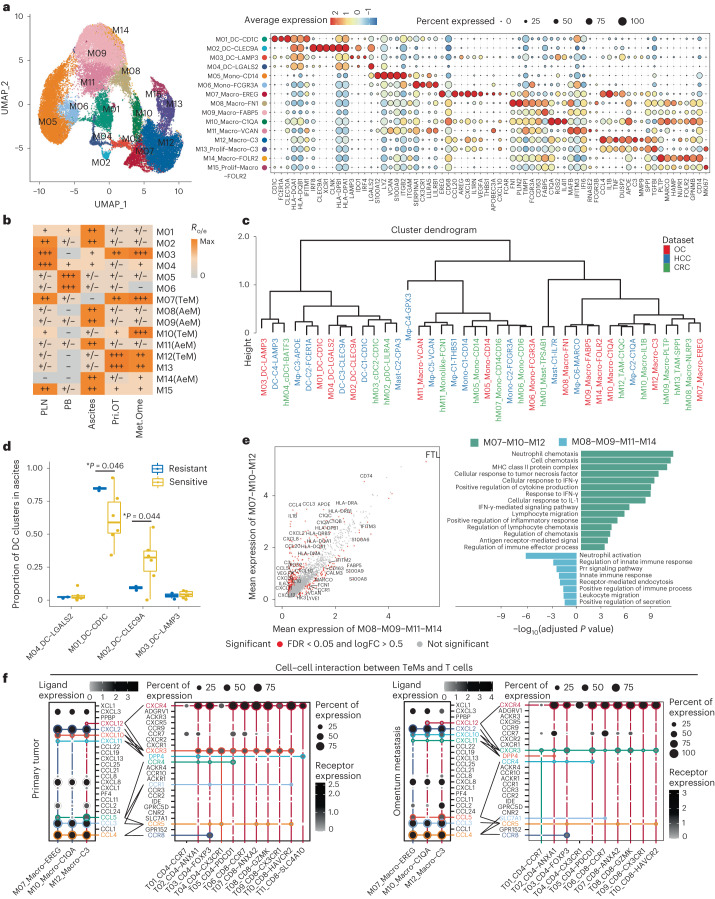
Two distinct functional states of tumor-enriched and ascites-enriched macrophages in HGSOC. **a**, UMAP projection of 15 myeloid clusters colored by clusters (left) and heat map showing expression patterns of selected genes across indicated clusters (right). **b**, Tissue preference of each myeloid cluster estimated by the R_o/e_. **c**, Hierarchical clustering comparing the similarity of myeloid cell clusters in our dataset (OC) with those reported in CRC and HCC. Clusters were colored by dataset. *n* = 3 tumor types were used for analysis. **d**, Frequency of DC subclusters as a proportion of all DCs in ascites from *n* = 6 platinum-sensitive patients and *n* = 2 platinum-resistant patients. Center line indicates the median value, bottom and top hinges represent the 25th and 75th percentiles, respectively and whiskers denote 1.5 × interquartile range. *P < 0.05, **P < 0.01, ***P < 0.001; two-sided *t*-test. **e**, Differentially expressed genes between TeMs (M07, M10 and M12) and AeMs (M08, M09, M11 and M14) (left). *P* value < 0.05; two-sided Wilcoxon test adjusted by the Benjamini–Hochberg (BH) procedure; log_2_(FC) > 0.5. *n* = 10 primary tumor, *n* = 4 matched omentum metastatic tumor and *n* = 8 ascites samples from ten patients with HGSOC were used for analysis. IFN, interferon; FDR, false discovery rate; FC, fold change. **f**, Dot plot showing the mean interaction strength for selected ligand–receptor pairs among macrophages and T cell clusters in tumors. Dot size indicates percentage of ligand–receptor expression in cells of one cluster, colored by average ligand–receptor expression level. *n* = 10 primary tumor and *n* = 4 matched omentum metastatic tumor from ten patients with HGSOC were used for analysis. For **a**,**b**, data were summarized from all *n* = 31 HGSOC samples. Source data

**Extended Data Fig. 6:**
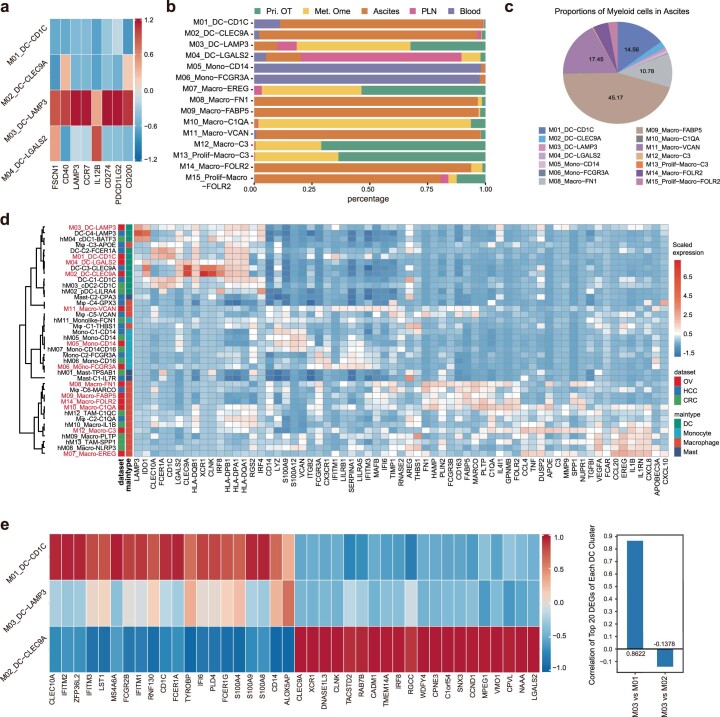
Clustering and characterization of myeloid cells, especially DC. **a**, Heatmap showing expression levels of cell maturation and immunoregulatory genes in 4 DC clusters. Rows represent clusters and columns represent genes. **b**, Tissue distribution of each myeloid cluster, colored by different tissues. **c**, Proportion of each myeloid cluster in all myeloid cells in ascites, colored by clusters. All n = 8 ascites samples from 10 HGSOC patients were analyzed. **d**, All-by-all heat map showing different gene expression in myeloid cells from datasets of our study (OV) and that of HCC and CRC (excluding all proliferative subsets). Clustered by similarities between myeloid subsets. Rows represent clusters and columns represent genes. N = 3 tumor types were used for analysis. **e**, Heatmap showing expression levels of top 20 differentially expressed genes of M01 and M02 in all three DC clusters (left), and correlation of DC-LAMP3 (M03) with DC-CD1C (M01) and DC-CLEC9A (M02) calculated using these genes (right). Genes, P value < 0.05, Two-sided Wilcoxon test adjusted by Benjamini-Hochberg (BH) procedure; log2(fold change) > 0.5. Correlation was analyzed using a Pearson correlation coefficient. For **a-b** and **e**, all n = 31 HGSOC samples were used for analysis.

For myeloid cells, unsupervised clustering gave rise to 15 clusters with distinct gene signatures (Fig. 4a). HLA^hi^CD14^−^ DC subsets (M01–M04) were further distinguished as *CD1C*^+^ DCs (cDC2), *CLEC9A*^+^ DCs (cDC1), *LAMP3*^+^ DCs and *LGALS2*^+^ DCs. Notably, the *LAMP3*^+^ DC cluster was also annotated as ‘mregDC’ for its high expression of maturation and immunoregulatory marker genes (such as *CCR7*, *IL12B*, *CD274*, *PDCD1LG2* and *LAMP3*), a cellular state induced upon uptake of tumor antigens^26^ (Extended Data Fig. 6a). In line with the tissue distribution patterns reported in other cancer types^27^, *LAMP3*^+^ DCs showed relatively comparable enrichment in tumor and lymph nodes. As *LAMP3*^+^ DCs exhibited increased expression of genes encoding a co-stimulatory molecule such as *CD40*, which is associated with interaction between myeloid cells and T cells^28^, and *IL12B*, which promotes T_H_1 development^29^(Extended Data Fig. 6a), we speculate that *LAMP3*^+^ DCs might also help potentiate the infiltration and differentiation of T_H_1-like cells in ovarian tumors. This could explain the higher enrichment indexes of both *LAMP3*^+^ DCs and T_H_1-like T cells in Met.Ome than in Pri.OT (Fig. 4b). Notably, we did not detect many conventional DCs (cDCs) in tumor tissues as reported in recent studies^27^, but instead observed their specific relative enrichment in malignant ascites (Fig. 4b and Extended Data Fig. 6b,c). To further elucidate the functions and relationships between different myeloid clusters, we performed similarity analysis of myeloid cells in our dataset with those reported in colorectal cancer (CRC)^28^ and hepatocellular carcinoma (HCC)^27^ (Fig. 4c). As expected, both cDC1 and cDC2 from different cancer types or tissue sources were clustered together, indicating their conserved phenotypes (Fig. 4c and Extended Data Fig. 6d). We also checked the potential origins of *LAMP3*^+^ DC in tumor and observed more cDC2-derived *LAMP3*^+^ DC (Extended Data Fig. 6e), which could be associated with higher proportions of cDC2 in ascites.

In addition, we noticed that the distribution of DC clusters was correlated with chemotherapy responses. Notably, among all DCs, the proportion of M01_DC-CD1C (cDC2) significantly increased in ascites of nonresponsive patients, whereas the M02_DC-CLEC9A (cDC1) proportion decreased (Fig. 4d). Although the previous studies reported that the protumor or antitumor responses of cDCs are uncertain among various types of tumors^30^, our observations indicated that cDC1 and cDC2 cells in the OC ascites might function in an opposite fashion in responses to platinum-based chemotherapy, which remains to be confirmed by further studies.

### Tumor-enriched and ascites-enriched macrophages

As for the monocyte/macrophage compartment, two blood-enriched clusters (M05 and M06) were characterized as *CD14*^+^ monocyte and *FCGR3A*^+^ nonclassical monocytes, respectively. The remaining clusters were all identified as macrophages (M07–M15) based on the high expression of *CD68* (Fig. 4a). Notably, macrophages detected in tumor and ascites were clustered primarily by their tissue distribution. Excluding the proliferating macrophages (M13 and M15), clusters showing relatively comparable enrichment in tumor sites (M07, M10 and M12) were denoted as tumor-enriched macrophages (TeMs), whereas the remaining clusters that showed relatively preferential enrichment in ascites (M08, M09, M11 and M14) were named as ascites-enriched macrophages (AeMs) (Fig. 4b). Among TeMs, *C3*^+^ M12 was the dominant subset distributed in both Pri.OT and Met.Ome, whereas *EREG*^+^ M07 and *C1QA*^+^ M10 tended to be enriched in Met.Ome. Likewise, four AeM subsets were further marked by their featured genes, leading to the classification of *FN1*^+^ M08, *FABP5*^+^ M09, *VCAN*^+^ M11 and *FOLR2*^+^ M14.

To further understand the heterogeneity of macrophage subsets across different tissues and tumor types, we also evaluated the similarities between macrophage clusters in our study and those reported in HCC and CRC, as mentioned above. *C3*^+^ TeMs (M12) and *C1QA*^+^ TeMs (M10) were clustered into the same branch, resembling the *IL1B*^+^ macro and *C1QC*^+^ TAMs identified in colon cancer, respectively (Fig. 4c). These clusters highly expressed *C1QA* and major histocompatibility complex (MHC) class II molecules associated with antigen presentation (Fig. 4a and Extended Data Fig. 6d). Notably, *C3*^+^ TeMs not only expressed genes related to phagocytosis and inflammation (*C3*, *CCL4* and *TNF*)^31^, but also upregulated transcriptomic programs associated with the response to tumors (*APOE*, *SPP1* and *TGFBI*)^32,33^ (Fig. 4a and Extended Data Fig. 6d), which was distinct from the *IL1B*^+^ macro in CRC^28^. Conversely, *EREG*^+^ TeMs (M07) exhibited high expression of chemokines like *CCL20*, *CCL4*, *CXCL10*, *CXCL8* and angiogenesis-related gene *VEGFA*, as well as low expressions of HLA-related genes, resembling the *SPP1*^+^ TAM identified in CRC (Fig. 4a,c and Extended Data Fig. 6d). Among AeM cells, *FABP5*^+^ AeM (M09), *FOLR2*^+^ AeM (M14) and *FN1*^+^ AeM (M08) were all clustered into the same branch with HCC ascites-enriched C6-MARCO, likely reflecting the environmental plasticity of macrophages. Of note, *VCAN*^+^ AeM (M11), characterized by high expression of transcripts associated with monocytes (*VCAN*, *S100A9* and *S100A12*)^34^, was clustered into the same branches with tumor-enriched C5-VCAN and ascites-enriched C1-THBS1 in HCC dataset and *FCN1*^+^ mono-like cells in CRC (Fig. 4c and Extended Data Fig. 6d). These two macrophages in HCC were defined as myeloid-derived suppressor cells (MDSCs) in the same differentiation lineage^27^. Therefore, *VCAN*^+^ AeMs (M11) in our study were more likely to be MDSCs distributed in ascites.

**Extended Data Fig. 7:**
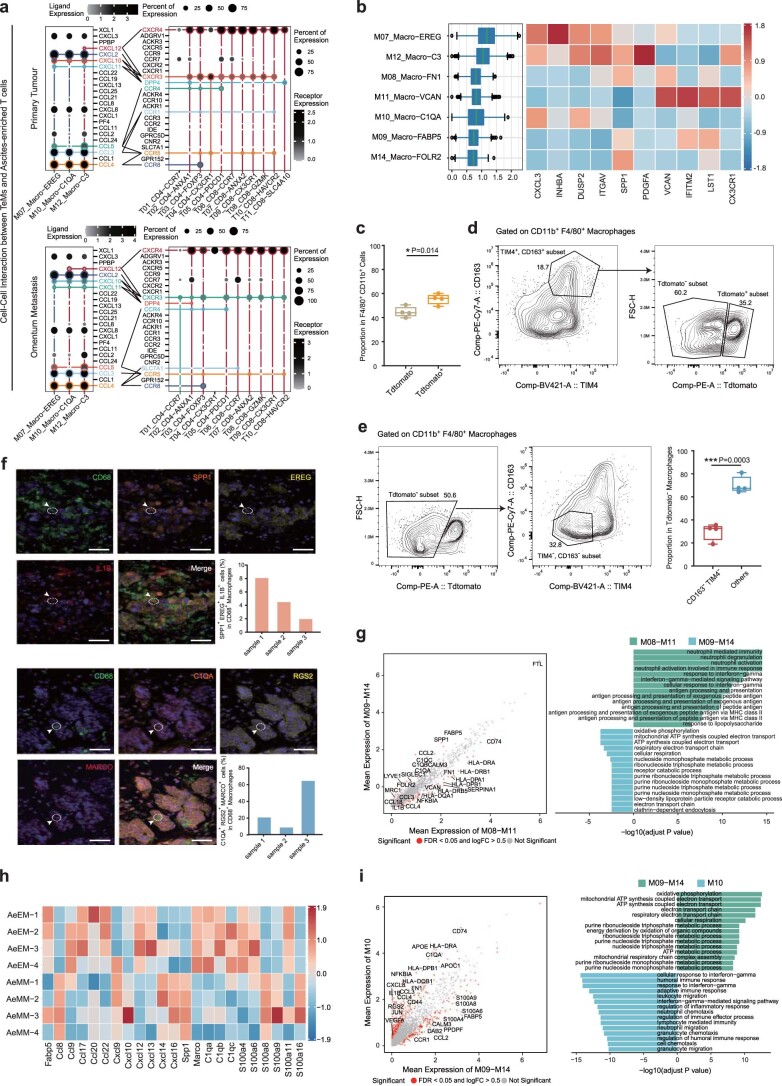
Different functions and ontogeny of macrophages enriched in tumors and ascites. **a**, Dot plot showing the mean interaction strength for selected ligand–receptor pairs among tumor-enriched macrophages and T cells in ascites. Dot size indicates percentage of ligand/receptor expression in cells of one cluster, colored by average expression levels. **b**, Expression levels of signature genes of monocyte-derived macrophages in 7 macrophage clusters. Center line indicates the median value, lower and upper hinges represent the 25th and 75th percentiles, respectively, and whiskers denote 1.5× interquartile range. **c**, Quantification of Tdtomato^−^or Tdtomato^+^ macrophages as a percentage of total CD11b^+^ F4/80^+^ macrophages using mouse ascites samples. **d**, Representative flow-cytometric plots showing frequency of Tdtomato^−^or Tdtomato^+^ cells in CD163^+^ TIM4^+^ RTMs using mouse ascites samples. **e**, Representative flow-cytometric plots, and summary of frequency of CD163^+^ or TIM4^+^ cells in Tdtomato^−^macrophages using mouse ascites samples. **f**, Representative examples of ovarian tumor stained by multicolored IHC (left) and the quantification plots (right). The upper panel indicates M07, and lower M10. Original magnification, 20x; scale bar, 50um. N = 3 individual patient tumors were examined independently per staining analysis. **g**, Differentially expressed genes (left) and differentially activated pathways (right) between tissue-resident macrophages (M09 and M14) versus monocyte-derived macrophages (M08 and M11) in ascites. **h**, Heatmap showing expression levels of indicated genes in macrophages of mouse ascites using samples from n = 4 mice. AeEM: ascites-enriched embryonic macrophage; AeMM: ascites-enriched monocyte-derived macrophage. **i**, Differentially expressed genes (left) and differentially activated pathways (right) between tissue-resident macrophages enriched in tumors (M10) versus RTMs enriched in ascites (M09 and M14). (**c** and **e**): Center line indicates the median value, lower and upper hinges represent the 25th and 75th percentiles, respectively, and whiskers indicates min to max. *P < 0.05, **P < 0.01, ***P < 0.001, unpaired two-sided t-test. (**g** and **i**): Genes, P value < 0.05, two-sided Wilcoxon test adjusted by Benjamini-Hochberg (BH) procedure; log2(fold change) > 0.5. Pathways, GO, adjusted P value by Benjamini-Hochberg (BH) procedure < 0.05. For **a-b**, **g** and **i**, all n = 31 HGSOC samples were used for analysis. For **c-e**, n = 4 independent experiments using 4 mouse ascites samples were used for analysis. Source data

We next investigated the different functional states of TeMs (M07, M10 and M12) and AeMs (M08, M09, M11 and M14). We observed that TeMs predominantly expressed MHC class II molecules and *CD74*, which are essential for antigen processing and presentation to CD4^+^ T cells. TeMs also upregulated the expressions of *VEGFA*, implying a role for tissue macrophages in promoting tumor angiogenesis. Moreover, we observed upregulated chemokines (such as *CCL3/4/5* and *CXCL10/11/12*) expression in TeMs (Fig. 4e), suggesting the importance of tumor macrophages in recruiting T cells^35,36^. Cell–cell interaction analysis within tumor tissues also confirmed that TeMs participated actively in the recruitment of T cells through CXCL10/11–CXCR3, CCL3/4/5–CCR5 and CXCL12–CXCR4 signaling (Fig. 4f). In primary tumors, *EREG*^+^ macro (M07) expressed increased levels of *CXCL10*/*11*, whereas *C3*^+^ macro (M12) highly expressed *CXCL12*; however, in metastatic tumors, it was surprising to find that the dominant source of *CXCL10*/*11* was switched from *EREG*^+^ M07 to *C3*^+^ M12 and *C1QA*^+^ M10 upregulated the expression level of *CXCL12*, indicating a reprogramming of macrophages in metastatic tumors. In addition, *EREG*^+^ TeM (M07) and *C3*^+^ TeM (M12) also showed preferential expression of molecule CCL4 and CCL5, which binds to CCR4 and CCR8, receptors highly expressed by CD4^+^ T_reg_ cells. We also found very similar interaction patterns between TeMs and ascites T cells (Extended Data Fig. 7a). Collectively, our data suggested the function of TeMs in recruiting T cells and shaping an immunosuppressive niche in tumors.

By contrast, AeMs exhibited high expression levels of *S100A* family (*S100A8* and *S100A9*) associated with tumor progression^37^ and relatively lower levels of *HLA-II* genes (Fig. 4e), indicative of a dysfunctional state of macrophages which further contributed to a protumor environment in ascites. Moreover, AeMs also showed strong enrichment of leukocyte migration pathway, with specifically upregulated expression level of *CCR1* (Fig. 4e). Notably, we also noticed that AeMs highly expressed *LYVE1* and *CD163* (Fig. 4e), signature genes of tissue-resident macrophages (RTMs) found in multiple human tissues^38^, implying that RTMs might be an important source of macrophages in ascites.

### Dichotomous ontogeny of TeMs and AeMs in OC

**Fig. 5:**
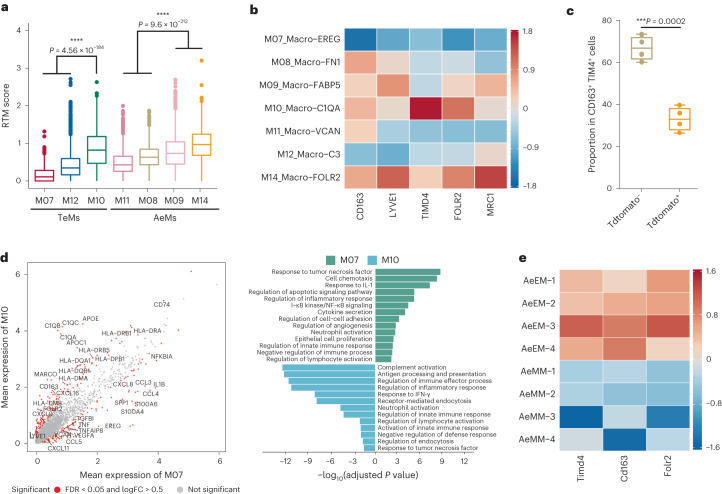
Two different origins of tumor-enriched and ascites-enriched macrophages in HGSOC. **a**, Bar plot showing the mean expression levels of tissue-resident marker genes in all macrophage clusters. Center line indicates the median value, lower and upper hinges represent the 25th and 75th percentiles, respectively and whiskers denote 1.5 × interquartile range. *P < 0.05, **P < 0.01, ***P < 0.001, two-sided *t*-test, adjusted by the BH procedure. **b**, Expression levels of tissue-resident relevant genes in seven macrophage clusters. Rows represent clusters and columns represent genes. **c**, Quantification of tdTomato^−^ or tdTomato^+^ macrophages as a percentage of total CD163^+^ TIM4^+^ RTMs in *n* = 4 independent experiments using *n* = 4 mice ascites samples, related to Extended Data Fig. 7d. Center line indicates the median value, bottom and top hinges represent the 25th and 75th percentiles, respectively and whiskers indicates min to max. *P < 0.05, **P < 0.01, ***P < 0.001, unpaired two-sided *t*-test. **d**, Differentially expressed genes (left) and differentially activated pathways (right) between tissue-resident macrophages (M10) versus monocyte-derived macrophages (M07) in tumor sites (left). Genes, *P* value < 0.05, two-sided Wilcoxon test adjusted by the BH procedure; log_2_(fold change) > 0.5. Pathways, Gene Ontology (GO), adjusted *P* value by the BH procedure <0.05. *n* = 10 primary tumor and *n* = 4 matched omentum metastatic tumor from ten patients with HGSOC were used for analysis. **e**, Heat map showing expression levels of tissue-resident marker genes in macrophages of mouse ascites using ascites samples from *n* = 4 mice. AeEM, ascites-enriched embryonic macrophage; AeMM, ascites-enriched monocyte-derived macrophage. Rows represent repetitive samples and columns represent genes. For **a**,**b**, data were summarized from all *n* = 31 HGSOC samples. Source data

Recent studies in mice have suggested that tumor-associated macrophages could have both RTM and monocyte origins^39^. Here, to further infer the ontogeny of TeMs and AeMs, we defined an RTM score using a set of tissue-resident relevant genes, including *CD163*, *LYVE1*, *FOLR2*, *MRC1* and *TIMD4* (Fig. 5a,b)^39–41^. Two of three TeM subsets (M07 and M12) showed much lower RTM scores compared to M10, whereas about half of cells from AeM clusters (M09 and M14) had relatively higher RTM scores (Fig. 5a). Additionally, a set of monocyte-derived macrophage-associated genes were used to complement the analysis of macrophage origins. The results displayed a similar trend, with M07 and M12 exhibiting the highest potential of monocyte-derived ontogeny (Extended Data Fig. 7b)^28,42^. These findings implied that macrophages identified in OC had two possible origins, with monocyte-derived macrophages as the dominant components in tumors and RTMs accounting for a large part in ascites-enriched subsets. As reported, although RTMs in adult tissues are gradually replaced by circulating monocytes, there constantly exists a self-maintenance population of RTMs arising from embryonic precursors^43^. To explore the extent to which embryonic peritoneal macrophages contribute to ascites-enriched RTMs, we employed Ms4a3^Cre^-Rosa^TdT^ monocyte fate-mapping mouse models^42^ to precisely quantify the different ontogeny of macrophages in malignant ascites of ovarian tumor-bearing mouse. Based on the flow cytometry data, nearly half of the AeMs were embryonic-derived macrophages with ~45% proportion of tdTomato^−^ cells (Extended Data Fig. 7c). Further, ~70% CD163^+^TIM4^+^ RTMs in malignant ascites were contributed by embryonic precursors (Fig. 5c and Extended Data Fig. 7d,e). These results implied that embryonic macrophages as an important resource of AeMs, contributing to the maintenance of RTMs in the peritoneal microenvironment in OC.

Subsequently, we characterized the distinct signatures of TeMs or AeMs with divergent ontogeny. RTM-derived M10 expressed significantly higher levels of complement *C1Q* genes and HLA-II related genes (*HLA-DRA*, *HLA-DPB1* and *HLA-DQA1*) (Fig. 5d). By contrast, monocyte-derived M07 showed specific expression of *VEGFA*, *IL1B* and *TNF*. The pathway analysis also revealed a strong enrichment of complement activation and antigen processing and presentation pathways in RTM-derived M10, whereas tumor angiogenesis, response to IL-1 and NF-κB pathways were significantly increased in monocyte-derived M07 (Fig. 5d). Multicolor imaging data further confirmed the coexistence of monocyte-derived M07 *EREG*^+^ macro and RTM-derived M10 *C1QA*^+^ macro in ovarian tumors (Extended Data Fig. 7f). Next, we compared the distinct biological features of ascites-enriched RTMs (M09 and M14) and monocyte-derived AeMs (M08 and M11). RTMs in ascites exhibited higher expression levels of complement *C1Q* genes (Extended Data Fig. 7g), consistent with the tumor-enriched RTMs. Besides, ascites-enriched RTMs expressed specifically increased levels of *FABP5*, associated with tumor regulation^44^ and CCL2 molecule responsible for monocyte recruitment (Extended Data Fig. 7g). Bulk RNA sequencing of tumor-bearing fate-mapping mice models also confirmed the upregulation of *C1q* genes, *Fabp5* and RTM signature genes, including *Timd4* and *Cd163* in ascites-enriched embryonic macrophages (Fig. 5e and Extended Data Fig. 7h), further confirming that embryonic macrophages might be a major source of RTMs in the ascites of patients with OC. Of note, we observed that ascites-enriched RTMs expressed lower levels of *CD74* and HLA-II related genes than monocyte-derived AeMs, contrary to the observations of TeMs (Extended Data Fig. 7g), likely reflecting the different ontogeny of RTMs in ascites and tumor tissues. Furthermore, we compared the differences between RTMs distributed in tumor and ascites. Ascites-enriched RTMs (M09 and M14) exhibited specific enrichment of oxidative phosphorylation and metabolic-related pathways, whereas tumor-enriched RTMs (M10) significantly upregulated immune response and immune cell migration pathways (Extended Data Fig. 7i). Notably, RTMs in ascites also showed specific high expression of CCL2, which mediates the recruitment of *CCR2*^+^ monocytes^45^. Taken together, our analyses establish the connections between macrophage ontogeny-specific features and their various functions in tumor growth. Further studies will be needed to fully discriminate macrophage ontogeny and to attribute the specific functional profile of these macrophages to their ontogenies.

### Stromal cells contribute to shaping the ascites TME

**Fig. 6:**
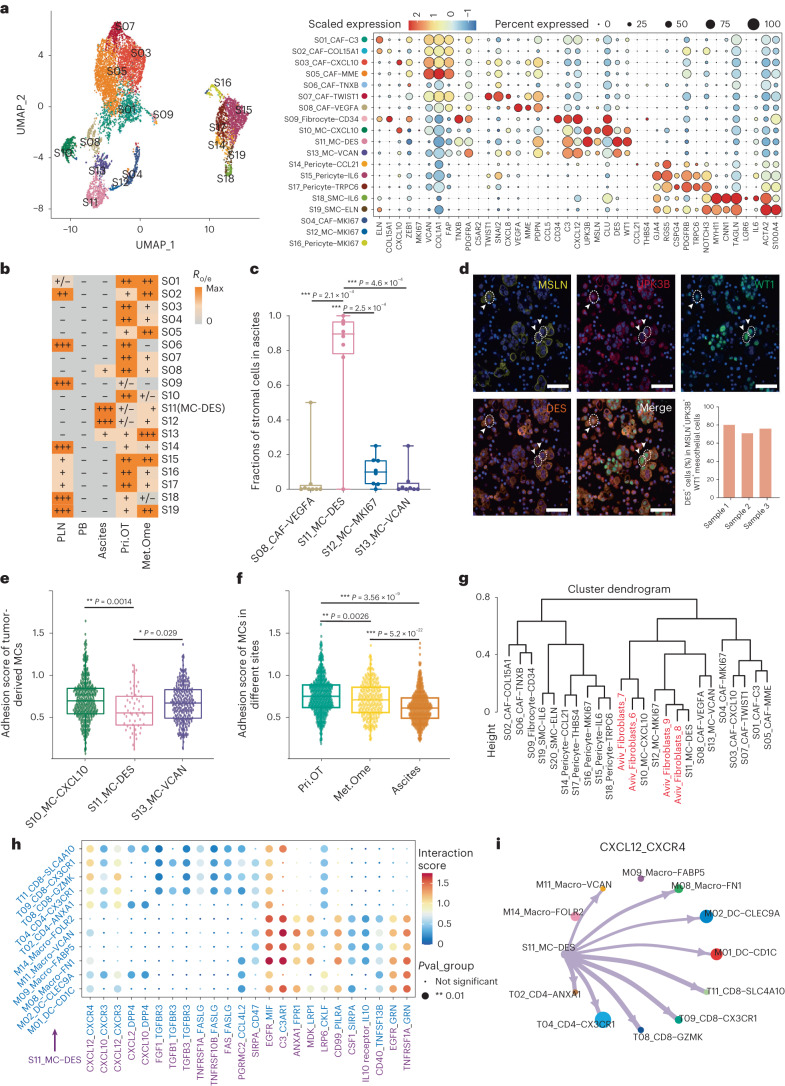
Characterization of stromal cell clusters of HGSOC, especially *DES*^+^ mesothelial cells in ascites. **a**, UMAP projection of 19 stromal cell clusters colored by clusters (left) and heat map showing expressions of selected genes across indicated clusters (right). **b**, Tissue preference of each stromal cell cluster estimated by the ratio of observed to expected cell numbers (R_o/e_). **c**, Frequency of each ascites-enriched stromal cell cluster as a proportion of all stromal cells in ascites, *n* = 8 ascites samples were analyzed. Center line indicates the median value, bottom and top hinges represent the 25th and 75th percentiles, respectively and whiskers indicates min to max. *P < 0.05, **P < 0.01, ***P < 0.001, unpaired two-sided *t*-test. **d**, Representative example of ascites cell precipitation from one patient with HGSOC stained by multicolored immunohistochemistry and the corresponding quantification plot. Original magnification, ×20; scale bar, 50 μm. *n* = 3 individual patient samples were examined independently. **e**,**f**, Bar plots showing the geometric mean expression levels of adhesion-associated genes in three mesothelial cell clusters from a total of *n* = 14 HGSOC tumor samples (**e**) or in all mesothelial cells in *n* = 10 primary tumor, *n* = 4 omentum metastasis and *n* = 8 ascites from ten patients with HGSOC, respectively (**f**). Center line indicates the median value, bottom and top hinges represent the 25th and 75th percentiles, respectively and whiskers denote 1.5 × interquartile range. *P < 0.05, **P < 0.01, ***P < 0.001, two-sided Wilcoxon test. Each dot corresponds to a single cell. **g**, Hierarchical clustering comparing the similarity of stromal cell clusters in our dataset with those reported in OC ascites by Aviv. The clusters in black font were detected in our dataset. **h**, Bubble heat map showing the mean interaction strength for selected ligand–receptor pairs between *DES*^+^ mesothelial cells and various immune cell clusters. Dot size indicates *P* value generated by permutation test, colored by interaction strength levels. *DES*^+^ MCs were cells providing ligands. **i**, Chord diagram showing predicted cell–cell interactions of CXCL12*–*CXCR4 ligand pair between *DES*^+^ mesothelial cells and various immune cell clusters in ascites. The arrow width indicates the interaction strength levels. For **a**,**b**,**h**,**i**, all *n* = 31 HGSOC samples were analyzed. Source data

**Extended Data Fig. 8:**
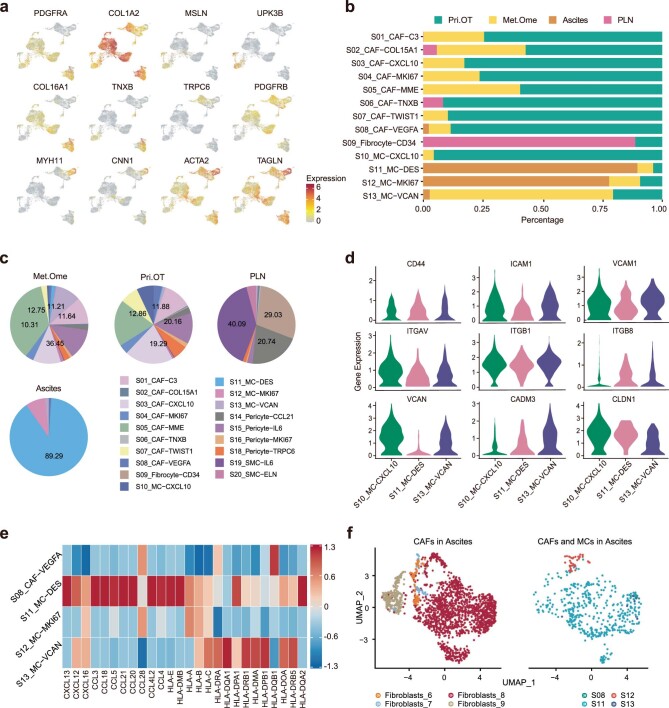
Basic information of stromal cell clusters and gene expressions of mesothelial cells in ascites. **a**, UMAP plots showing expression levels of cluster-specific marker genes in stromal cells. **b-c**, Tissue distribution of each stromal cell cluster, colored by tissues (**b**) and clusters (**c**). Stromal cells were un-detectable in blood. **d**, Violin plots showing the expression levels of adhesion-associated genes in 3 mesothelial cell clusters derived from tumor sites. Totally n = 14 HGSOC tumor samples, including n = 10 primary tumor and n = 4 omentum metastasis samples were analyzed. **e**, Heatmap showing the expression levels of chemokines and HLA-related genes in 4 stromal cell clusters detected in ascites. Rows represent clusters and columns represent genes. All n = 8 ascites samples from HGSOC patients were used for analysis. **f**, UMAP plots showing the similarity of ascites-derived cancer-associated fibroblasts (CAF) reported by Aviv (left) and stromal cells enriched in ascites in our dataset (right). For **a-c**, all n = 31 HGSOC samples were used for analysis.

For nonimmune cells, we first dissected the gene signatures and tissue distributions of all 19 stromal clusters revealed in this study (Fig. 6a,b and Extended Data Fig. 8a–c), including 9 fibroblast clusters (*COL1A2*^+^ *PDGFRA*^+^), 4 mesothelial cell (MC) clusters (*MSLN*^+^*UPK3B*^+^), 4 clusters of pericytes (*CSPG4*^+^*TRPC6*^+^) and 2 vascular smooth muscle cell clusters (*MYH11*^+^*CNN1*^+^)^46,47^. Among MCs, *DES*^+^ MC (S11) was the dominant stromal cluster in ascites (Fig. 6b,c), which was confirmed by multicolor immunohistochemistry (Fig. 6d). In contrast, *VCAN*^+^ MCs (S13) were highly enriched in Met.Ome (Fig. 6b and Extended Data Fig. 8b,c). It has been shown previously that MCs undergo morphological changes and detach from the peritoneal surface during OC peritoneal metastases^16^. We therefore compared the expression levels of cell-adhesion-associated genes (*CD44*, *ICAM1*, *ITGAV*, *ITGB1*, *ITGB8*, *VCAM1*, *VCAN*, *CADM3* and *CLDN1*) in tumor-derived MCs and found the lowest expression in *DES*^+^ MCs (Fig. 6e and Extended Data Fig. 8d), suggesting that *DES*^+^ MCs were more likely to fall off into the ascites from tumor tissues. Meanwhile, we observed a significantly decreased cell adhesion potential of MCs in Met.Ome compared to that in Pri.OT (Fig. 6f). These analyses indicated that the loss of cell–cell adhesions could be a reason for MCs to shed from the omentum into ascites, which provides a favorable condition for tumor cell metastasis and colonization.

Notably, *DES*^+^ MCs showed high expression of *CXCL12, CXCL13* and *CXCL16* (Extended Data Fig. 8e), reminiscent of the recently reported immunomodulatory cancer-associated fibroblasts (CAFs) identified in ascites^15^. By integrating our dataset with that of CAFs in OC ascites, we further confirmed the similarities between *DES*^+^ MCs in our study and the immunomodulatory CAFs^15^ (Fig. 6g and Extended Data Fig. 8f). We also observed that *DES*^+^ MCs had high potential to extensively interact with memory T cells and macrophages (Fig. 6h,i). One of the significantly enriched ligand–receptor pairs was CXCL12–CXCR4, which is associated with recruitment of immune cells^48^. This could help explain the underlying reasons for the abundance of immune cells in ascites and the inflammatory milieu of ascites. *DES*^+^ MCs were also predicted to interact with macrophages and MAIT cells via C3-C3AR1 (ref. ^49^), which would lead to the further recruitment of these cells to enhance the inflammatory response in ascites (Fig. 6h). Taken together, the results indicate that *DES*^+^ MCs might constitute a key cellular component that plays an important role in the regulation of inflammatory and immune responses in OC ascites.

### Endothelial cell phenotypes associated with chemotherapy response

**Fig. 7:**
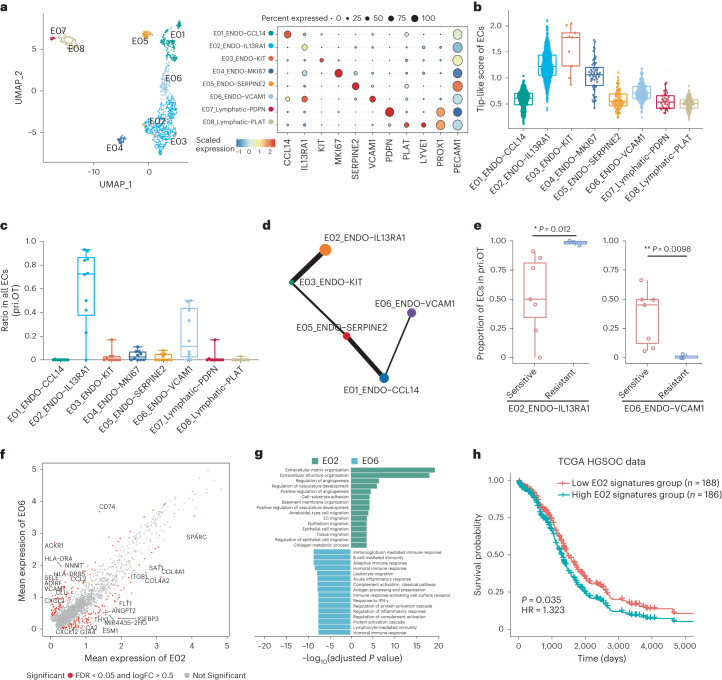
Characterization of endothelial cell phenotypes within two tumor sites in HGSOC. **a**, UMAP projection of eight endothelial cell clusters colored by clusters (left) and heat map showing expression patterns of selected genes across indicated clusters (right). **b**, Bar plot showing the geometric mean expression levels of tip-like genes (referred to in Extended Data Fig. 9b) in eight endothelial cell (EC) clusters. Each dot corresponds to a single cell. Center line indicates the median value, bottom and top hinges represent the 25th and 75th percentiles, respectively and whiskers denote 1.5 × interquartile range. **c**, Frequency of each endothelial cluster as a proportion of all endothelial cells in *n* = 10 primary tumor samples from ten patients with HGSOC. The center line indicates the median value, bottom and top hinges represent the 25th and 75th percentiles, respectively and whiskers indicates min to max. Each dot corresponds to one sample. **d**, PAGA analysis of endothelial cells. Each dot represents a cell cluster. **e**, Frequency of E02 (left) and E06 (right) cluster as a proportion of all endothelial cells in ten primary tumor samples from *n* = 7 platinum-sensitive and *n* = 3 platinum-resistant patients. Center line indicates the median value, bottom and top hinges represent the 25th and 75th percentiles, respectively and whiskers denote 1.5 × interquartile range. *P < 0.05, **P < 0.01, ***P < 0.001; two-sided *t*-test. **f**, Differentially expressed genes between E02 and E06 cluster. *P* value < 0.05; two-sided Wilcoxon test adjusted by the BH procedure; log_2_(FC) > 0.5. **g**, Differentially activated pathways between E02 and E06 cluster. GO, adjusted *P* value by the BH procedure <0.05. **h**, The Kaplan–Meier overall survival curves of patients with HGSOC grouped by the gene signature expression of *IL13RA1*^+^ ENDO cells. HR, hazard ratio. Multivariate Cox regression. *P* value was determined by Kaplan–Meier survival curves and log-rank test. For **a**,**b**,**d**,**f**,**g**, all *n* = 31 samples from ten patients with HGSOC were used for analysis. Source data

**Extended Data Fig. 9:**
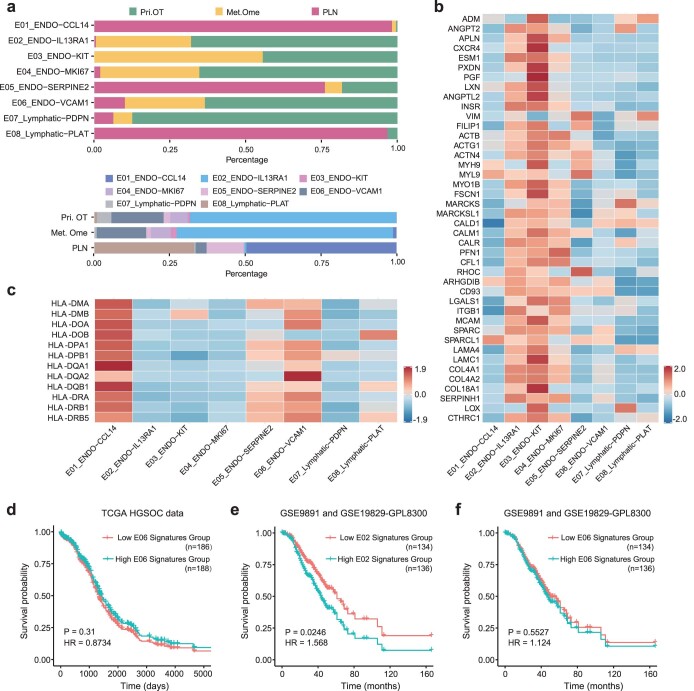
Distribution and gene expressions of endothelial cells as well as association of E02, E06 clusters with HGSOC prognosis. **a**, Tissue distribution of each endothelial cell cluster identified in HGSOC patients, colored by different tissues (upper) and clusters (lower). **b**, Heatmap showing the expression levels of indicated marker genes of tip-like endothelial cell in 8 endothelial cell clusters. Rows represent clusters and columns represent genes. **c**, Heatmap showing the expression levels of genes involved in MHC-II antigen presentation in 8 endothelial cell clusters. Rows represent clusters and columns represent genes. **d**, The Kaplan–Meier overall survival curves of HGSOC patients grouped by the gene signature expression of *VCAM1*^+^ ENDO cells (E06). TCGA OV data. HR, hazard ratio. Multivariate Cox regression. **e**, The Kaplan–Meier overall survival curves of HGSOC patients grouped by the gene signature expression of *IL13RA1*^+^ ENDO cells (E02). TCGA OV data. HR, hazard ratio. Multivariate Cox regression. **f**, The Kaplan–Meier overall survival curves of HGSOC patients grouped by the gene signature expression of *VCAM1*^+^ ENDO cells (E06). Microarray GSE9891 and GSE19829-GPL8300. HR, hazard ratio. Multivariate Cox regression. Statistical significance (P value) was determined by Kaplan–Meier survival curves and log-rank test **(d-f)**. For **a-c**, all n = 31 HGSOC samples were used for analysis.

Among all endothelial cells, E07 and E08 were annotated as lymphatic endothelial cells based on the expression of canonical marker *PROX1* (ref. ^50^), whereas other clusters were identified as vascular endothelium (Fig. 7a and Extended Data Fig. 9a). It has been reported that tumor angiogenesis mainly undergoes two alternate processes, including vessel sprouting by migrating tip endothelial cells and sprout elongating^51^, suggesting that the tip cells could accelerate angiogenesis whereas other endothelial cells were relatively more static. Here, cluster E03 showed high expression of genes associated with endothelial cell migration and matrix remodeling^50^ (Fig. 7b and Extended Data Fig. 9b), resembling the tip cells detected in lung tumor, which indicated poor prognosis of patients^50^.

Further deciphering the transcriptional trajectories of endothelial cells using PAGA, we found that *IL13RA1*^+^ E02 and *VCAM1*^+^ E06, two major endothelial cell clusters in tumor tissues, exhibited unique features (Fig. 7c,d). We observed that *IL13RA1*^+^ E02 showed closer connectivity with the tip-like cells (E03) and upregulated tip cells signatures, whereas *VCAM1*^+^ E06 were positioned at another branch (Fig. 7b,d and Extended Data Fig. 9b). Notably, the proportion of *IL13RA1*^+^ E02 was significantly increased in Pri.OT samples of nonresponsive patients, whereas *VCAM1*^+^ E06 was depleted in Pri.OT samples of platinum-resistant patients (Fig. 7e). Moreover, *IL13RA1*^+^ E02 expressed higher levels of *SPARC*, *COL4A1*, *COL4A2*, *ANGPT2* and *ITGB1* (Fig. 7f), genes involved in vasculature development, epithelial cell proliferation and migration pathways (Fig. 7g), suggesting that *IL13RA1*^+^ E02 could contribute to chemotherapy resistance by promoting tumor angiogenesis and migration. In contrast, *VCAM1*^+^ E06 showed preferential expression of HLA-II related molecules and *ACKR1*, a marker of venular endothelium and with a known role in adhesive leukocyte-endothelial interactions^52^ (Fig. 7f,g), indicating that *VCAM1*^+^ E06 might assist lymphocytes infiltration and participate in antigen processing and presentation to enhance the chemotherapy sensitivity. Therefore, we hypothesized that the relative proportions of *IL13RA1*^+^ versus *VCAM1*^+^ endothelial cells might serve as a biomarker to predict the benefit from chemotherapy. Furthermore, we also examined whether *IL13RA1*^+^ and *VCAM1*^+^ endothelial clusters were associated with the long-term prognosis of HGSOC patients using data from The Cancer Genome Atlas (TCGA). We found that patients highly expressing the top 20 signature genes of *IL13RA1*^+^ E02 had shorter overall survival (Fig. 7h), further confirming their functions in tumor angiogenesis; however, signature genes of *VCAM1*^+^ E06 were not significantly correlated with clinical outcomes of patients with HGSOC (Extended Data Fig. 9d). We also used another independent microarray dataset to validate these results (Extended Data Fig. 9e,f).

### MAIT in ascites as potential predictors of platinum response

**Fig. 8:**
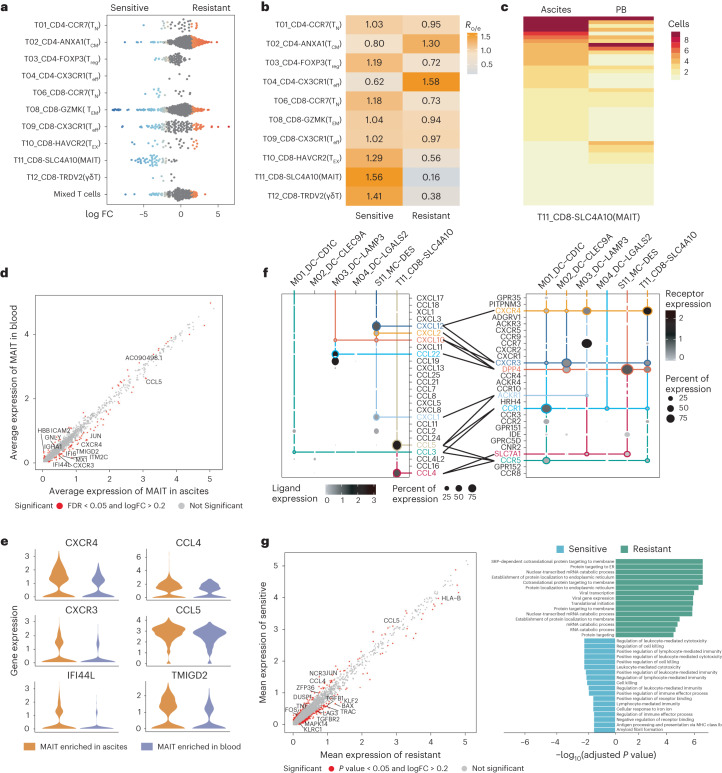
MAIT cells in ascites predict the chemotherapy efficacy of patients with HGSOC. **a**, UMAP plot showing the distribution preference of MAIT cells in eight ascites samples from *n* = 6 platinum-responsive and *n* = 2 nonresponsive patients as calculated by Milo. Each dot represents a single cell. **b**, The treatment-sensitivity preference (responsive or nonresponsive to platinum-based chemotherapy) of each T cell cluster estimated by R_o/e_ score. *n* = 8 ascites samples from *n* = 6 platinum-responsive and *n* = 2 nonresponsive patients with HGSOC were used for analysis. **c**, The distribution of clonal clonotypes within the MAIT cluster in ascites and PB. Each row represents an individual clonotype. **d**, Volcano plot showing differentially expressed genes between MAIT cells in ascites versus PB. Genes, *P* value < 0.05, two-sided Wilcoxon test adjusted by the BH procedure; log_2_(FC) > 0.2. **e**, Violin plots showing the expression levels of selected genes in MAIT cells derived from ascites and PB. **f**, Dot plot showing the mean interaction strength for selected ligand–receptor pairs among major immune and stromal cell clusters in ascites. *n* = 8 HGSOC ascites samples were analyzed. Dot size indicates percentage of ligand–receptor expression in cells of one cluster, colored by average ligand–receptor expression levels. **g**, Differentially expressed genes (left) and differentially activated pathways (right) between ascites-derived MAIT cells of *n* = 6 responsive versus *n* = 2 nonresponsive patients with HGSOC. SRP, signal recognition particle; ER, endoplasmic reticulum. Genes, *P* value < 0.05, two-sided Wilcoxon test adjusted by the BH procedure; log_2_(FC) > 0.2. Pathways, GO, adjusted *P* value by the BH procedure < 0.05. For **c**–**e**, all *n* = 8 ascites sample and *n* = 5 blood samples from patients with HGSOC were used for analysis. Source data

It has been reported that ascites accumulated in patients with OC is associated with chemotherapy response and prognosis^5^. Here, we further investigated the distinct compositions of the ascites microenvironment between responsive and nonresponsive patients. Based on the linear model analysis of all ascites-derived T cells using Milo, we noticed that MAIT cells were highly enriched in ascites of responsive patients before therapy, which was supported by the R_o/e_ data (Fig. 8a,b). It has been reported that MAIT cells could accumulate and function in the peritoneal cavity during a pathological process or in the tumor tissues^53,54^. In our study, MAIT cells were mainly detected in PB and ascites (Fig. 2b). We were able to detect 50 unique shared TCR clones between ascites- and blood-derived MAIT cells (Fig. 8c), suggesting PB as a potential source of ascites MAIT cells. Moreover, ascites-enriched MAIT cells upregulated homing receptors CXCR3 and CXCR4, which bind to CXCL12 and CXCL10, molecules upregulated by other ascites-enriched cells (such as cDC1 and *DES*^+^ MC) (Fig. 8d–f), further supporting the chemotaxis of MAIT cells. Ascites-enriched MAIT cells also showed preferential expression of genes related to cell activation (*TMIGD2*, *CCL4* and *CCL5*) (Fig. 8d,e), suggesting an activated status. We next compared the characteristics of ascites-enriched MAIT cells from responsive and nonresponsive patients. MAIT cells captured from responsive patients overexpressed genes associated with T cell activation, such as *ZFP36*, *JUN*, *DUSP1*, *NCR3* and *KLRB*^55–57^, whereas MAIT cells of nonresponsive patients highly expressed genes related to immunosuppression such as *LAG3* and *IFITM3* (Fig. 8g), suggesting that MAIT cells in ascites from patients with HGSOC with different responses to chemotherapy also exhibited different functions and phenotypes. Altogether, these results indicated that immune-activated MAIT cells might help patients benefit from chemotherapy, whereas MAIT cells in ascites of nonresponsive patients were more likely to be dysfunctional. Furthermore, the levels of activated MAIT cells in ascites could be a useful and noninvasive predictor of effective responses to chemotherapy.

## Discussion

Despite the usage of platinum-based chemotherapy and improved survival, most patients with advanced OC undergo relapse due to chemotherapy resistance^58^. Here, we applied scRNA-seq to five tissue types of 14 patients with OC with different sensitivities to chemotherapy and systematically dissected the complexity of TME as well as the connections among five tissues. Our analyses revealed that ascites-derived *GZMK*^+^ T_EM_, resembling the previously reported ‘pre-exhausted’ CD8^+^ T cells within tumors^11,13,25^, might be a major source of tumor-infiltrating T_EX_ cells. These findings suggest that ascites-derived memory T cells could migrate into tumor sites, acting as an additional important cell pool for TILs. As reported, pre-exhausted *GZMK*^+^ T subpopulation were regarded as pre-activated T cells which would accumulate in responsive lung cancer and melanoma tumors following immune-checkpoint-based treatment^11^. We suspected that accelerating the migration of ascites-derived *GZMK*^+^ T_EM_ cells into tumor sites could be a potential therapeutic strategy for OC. Moreover, we identified the proportions of MAIT cells in ascites as a potential predictive index in response to chemotherapy. Thus, our work on ascites-enriched T cells inspires us to rethink the functions of malignant ascites in shaping the tumor microenvironment. Future studies will be needed to fully understand the functional roles of these ascites T cells.

Here, we found that cDCs exhibited specific ascites-enriched distribution patterns in OC. We hypothesized that the presence of cDCs in ascites might serve as a potential source of *LAMP3*^+^ DCs in tumor tissues as we found in T cells, which require additional in vivo lineage-tracing validation. Moreover, it has been shown that macrophages were highly heterogeneous in the tumor TME^59^. We identified that macrophages of different origins and phenotypes coexisted within the ovarian tumor and ascites, with TeMs functioning in immune regulation and AeMs being more pro-inflammatory. RTMs in tumor tissues have been reported to provide a pro-tumorigenic niche in lung cancer and the omentum of ovarian tumors^60^. Our data also indicated the potential function of tumor regulation and monocyte recruitment of ascites-enriched RTMs.

Ultimately, we identified specific populations of stromal cells playing important roles in tumor progression, such as *DES*^+^ mesothelial cells in ascites and *IL13RA1*^+^ endothelial cells in tumor site. Our findings reveal that ascites-enriched *DES*^+^ MCs could help remold the microenvironment of ascites through recruiting T cells and macrophages via CXCL12–CXCR4. The chemokine CXCL12 is known to be expressed by CAFs and binds to the receptor CXCR4, mediating the recruitment of immune cells in tumors^13^. Further, *IL13RA1*^+^ endothelial cells exhibited tip-like signatures involved in angiogenesis and were significantly enriched in platinum-resistant patients. Navigating tip cells usually lead the way during vessel sprouting, which could facilitate tumor progression and implies a worse prognosis^50^. These observations suggest that the abundance of *IL13RA1*^+^ tip-like endothelial cells might activate angiogenesis and further influence chemotherapy resistance.

In conclusion, we depicted a comprehensive atlas of the OC microenvironment and revealed the connections between ascites and two tumor sites. Our work provided additional insights into the biological factors that help remodel the OC TME and identified specific cell subpopulations that might serve as potential predictive markers for chemotherapy and prognostic markers of long-term survival, as well as new therapeutic targets or strategies for overcoming platinum resistance and immune suppression.

## Methods

This study complies with all relevant ethical regulations and was approved by the Ethics Committee of Xinhua Hospital Affiliated to Shanghai Jiaotong University School of Medicine and Fudan University Shanghai Cancer Center. Written informed consent was provided by all participants.

### Human specimens

Fourteen patients pathologically diagnosed with OC were enrolled in this study for single-cell sequencing. None of the patients had an autoimmune disorder or a history of previous cancer. Only one patient diagnosed with undifferentiated OC was treated with adjuvant chemotherapy. The disease stages of these patients were classified according to the 2018 International Federation of Gynecology and Obstetrics staging system. Fresh samples including primary ovarian tumor, omentum metastatic tumor, PLNs, malignant ascites and PB were obtained from the patients during surgery. The patients received upfront debulking surgery followed by at least six courses of platinum-based chemotherapy. Platinum resistance was defined as progression within 6 months after the last treatment course. Patients HGSOC3, HGSOC6, HGSOC7 and ECO1 were platinum-resistant (nonresponsive), whereas the other patients, except UOC1 were platinum sensitive (responsive). Patients ranged in age from 43 to 82 years old, with a median age of 62 years. Five more patients pathologically diagnosed with HGSOC (patients HGSOC11–HGSOC15) were enrolled in this study for flow cytometry analysis of T cells. The available clinical metadata of these patients are summarized in Supplementary Table 1.

### In vivo mouse models

All animal experiments were approved by the Institutional Animal Care and Use Committee of the Model Animal Research Center, Xinhua Hospital, Shanghai Jiaotong University School of Medicine and were performed in compliance with the guidelines for the care and use of laboratory animals. The maximal tumor burden was not exceeded for mouse tumor experiments on the requirement of our ethics committee. All Ms4a3^TdT^ fate-mapping C57BL/6 mice were female and sourced from Florent Ginhoux Laboratories in Shanghai Institute of Immunology. All mice were provided with water and food and maintained in a pathogen-free facility (12-h light–dark cycle, room temperature at 20–4 °C and relative humidity kept at 45–65%) at the Model Animal Research Center, Xinhua Hospital, Shanghai Jiaotong University School of Medicine. Mice were given an intraperitoneal injection with 10^6^ ID8 cells in 500 µl sterile PBS (pH 7.4) to mimic the peritoneal spread of epithelial ovarian cancer when 4–5 weeks old. Details of cell lines are shown in Supplementary Table 6. For flow cytometry studies and bulk RNA-seq, bloody malignant ascites was collected 65 d after injection of tumor cells.

### ScRNA-seq data generation

Fresh tumor and lymph node samples were cut into approximately 1-mm^3^ pieces in RPMI-1640 medium (Invitrogen) with 10% fetal bovine serum (FBS; Gibco) and enzymatically digested with a MACS Tumor Dissociation kit (Miltenyi) for 30 min using a gentleMACS Octo Dissociator (Miltenyi) at 37 °C. Dissociated cells were subsequently passed through a 70-μm cell strainer (BD) and centrifuged at 400*g* for 10 min. The pelleted cells were then resuspended in red blood cell lysis buffer (Miltenyi) and incubated on ice for 5 min to lyse red blood cells. After washing twice with PBS (Invitrogen), cell pellets were resuspended in RPMI-1640 medium supplemented with 10% FBS. PB mononuclear cells were isolated using a leukocyte separation solution (Sigma-Aldrich) according to the manufacturer’s instructions. Malignant ascites samples were collected in 50-ml conical tubes (BD), followed by centrifugation for 10 min at 400*g*. The remaining pellet was washed twice with PBS and any residual red blood cells were lysed using the above-mentioned procedure. The concentration of single-cell suspensions was adjusted to about 500–1,200 cells per μl. Then, single-cell gene expression and immune repertoire measurements were conducted using the Chromium Single Cell V(D)J Reagent kit (10x Genomics) following the manufacturer’s instructions. All subsequent steps were performed following the standard manufacturer protocols. Completed libraries were sequenced on an Illumina NovaSeq6000 system.

### ScRNA-seq data processing

Low-quality cells were filtered out if cells had fewer than 200 genes expressed or >10% unique molecular identifiers (UMIs) linked to mitochondrial genes. The gene expression matrices of the remaining cells were generated with log normalization and linear regression using the NormalizeData and ScaleData functions of the Seurat package (v.3.1.4). Cells with expression of more than one major cell marker were considered as doublets and removed from each cluster individually. The remaining cells that passed the filtering criteria were considered single cells. We also identified 2,010 platelets with high expression of *pro-platelet basic protein*. Almost all platelets were found in PB mononuclear cell samples and they are not discussed in this study. For visualization, the dimensionality of each dataset was further reduced using UMAP with the Seurat function Run-UMAP. The principal components (PCs) used to calculate the embedding were the same as those used for clustering.

### Unsupervised clustering and identification of cell subpopulations

After the main cell populations were identified by first-run clustering, we ran the Seurat pipeline for a second time. Unwanted effects caused by percentage of mitochondrial UMI counts were removed by regression in this run. The selection of the resolution on the characteristics of each dataset and the top *n* PCs from principal-component analysis were used for identification of clusters. For T lymphocytes, we performed extra batch correction across different samples with Harmony (v.1.0) at the default settings. Small clustering groups with expression of dual-lineage signatures, including *EPCAM–PECAM1–CD3D*, *EPCAM–CD79A*, *PECAM1–CD79A* and *CD79A–CD3D*, were removed from downstream analysis. For other cell types, we did not conduct any batch correction as no obvious clustering bias using raw transcripts per million-like expression data would affect our downstream analyses. Supplementary Table 5, showing the distribution of cell subclusters in five tissues and patients with HGSOC, was provided as diagnostic data to ensure that none of the clusters would arise from individual tissues or patients.

### Identification and analysis of malignant cells with CNV estimation

Copy number variation (CNV) for individual cells was estimated using inferCNV (v.1.2.1) with a 100-gene sliding window. The method to use for smoothing was pyramidal. Genes with an average read count <0.1 among reference cells were filtered when running inferCNV. Endothelial cells, stromal cells, lymphoid cells and myeloid cells were used to define the reference. Epithelial cells were used for the observations. Downsampling was conducted for both the reference and observations to increase the speed of analysis. Epithelial cells were classified to malignant cells using a similar method previously described by Wu et al.^61^

### Tissue distribution of clusters

We calculated the R_o/e_ for each cluster in different tissues to quantify the tissue preference of each cluster^18,25^. The expected cell numbers for each combination of cell clusters and tissues were obtained from the chi-squared test. One cluster was identified as being enriched in a specific tissue if R_o/e_ > 1. For most clusters, we used the R_o/e_ index (+++, R_o/e_ > 3; ++, 1 < R_o/e_ ≤ 3; +, 0.2 ≤ R_o/e_ ≤ 1; +/−, 0 < R_o/e_ < 0.2; and −, R_o/e_ = 0) to define the cluster preference in a specific tissue. Furthermore, when analyzing the association between each T cell subset and treatment responses to platinum-based chemotherapy, we applied miloR (v.1.5.0), a differential abundance testing framework based on *K-*NN graphs and generalized linear models^62^.

### TCR analysis

The TCR sequences for each single T cell from 10x Genomics were processed using CellRanger (v.3.0.2) with the manufacturer-supplied human VDJ reference genome ‘GRCh38-alts-ensembl’. If two or more cells had the same identical α/β chain pair, the α/β chain pair were identified as clonal TCRs and these T cells were considered to originate from the same clonotypes, identified as clonal cells. After integrating TCR results with the gene expression data of 10x Genomics data, we identified TCR α/β-chain pairs for 59,334 cells. We then presented three STARTRAC indices to analyze different aspects of T cells based on paired single-cell transcriptomes and TCR sequences using STARTRAC (v.0.1.0) as previously described^18^. STARTRAC-expa, STARTRAC-migr and STARTRAC-tran are designed to measure the degree of clonal expansion, tissue migration and state transition of T cell clusters upon TCR tracking, respectively. The MAIT cells (T11) and γδT cells (T12) were not included in these types of analyses because they have distinct TCRs.

### Developmental trajectory inference

#### PAGA

To characterize the developmental origins of CD4^+^ and CD8^+^ T cells, respectively, we performed the partition-based graph abstraction method PAGA^23^, a part of the single-cell analysis package Scanpy (v.1.7.2) in Python (v.3.6.13)^63^, to infer the potential differential trajectory. Moreover, we used PAGA to assess the most likely trajectories of cell progression among endothelial cells in OC. The computations were carried out using default parameters. The edge connectivity between each subpopulation node for all edges are further compared by using an unpaired two-sided Student’s *t*-test.

#### Palantir

We also applied Palantir^24^ to complement the trajectory analysis using default parameters.

### Comparison dendrograms for similarity analysis of clusters

For an unsupervised comparison of the myeloid clusters identified from multiple datasets, we identified the top 2,000 highly variable genes across different clusters, calculated the mean expression of these genes in each cluster and performed hierarchical clustering using the distance defined as (1 − Pearson correlation coefficient)/2. Here, we used the batch-corrected expression value from the CCA function of the Seurat package. For comparison of stromal cell clusters reported in OC ascites^15^ and that detected in ascites in our study, we used the top 1,000 highly variable genes.

### Differential expression and Gene Ontology enrichment analysis

The significantly overexpressed marker genes for clusters were identified using the FindAllMarkers() function of Seurat. Genes with adjusted *P* value < 0.05 by Wilcoxon rank-sum test were defined as cluster-specific signature genes. For two different clusters, we used the Wilcoxon test to evaluate the significance of each gene, with multiple hypothesis correction using the BH procedure. Genes with adjusted *P* value <0.05 were considered as differentially expressed genes that were further used for GO enrichment analysis with the clusterProfiler package (v.3.14.3). GO terms with adjusted *P* values <0.05, using the BH procedure, were considered significant.

### RTM phenotype analysis

To identify the origins of macrophages enriched in tumors and ascites, we used a panel of genes associated with tissue-resident macrophages/monocytes to define the signature of macrophages in our study. The RTM/monocyte-like phenotype of each macrophage cluster was defined as the mean expression of gene signatures. *P* values were measured by two-sided *t*-test using Rstatix (v.0.7.0).

### Cell–cell interaction analysis

We used cellphoneDB (v.3.0.0)^64^ based on cellphoneDB database v.2.0.0 to infer cell–cell interactions of selected ligand–receptor pairs between tumor-enriched macrophages and T cell subsets, *DES*^+^ mesothelial cells and indicated immune cell subsets, as well as DC clusters and MAIT cells. The potential interaction strength between two cell subsets was predicted based on the expression of ligand–receptor pairs. The enriched ligand–receptor interactions between two cell subsets were calculated based on a permutation test. We extracted significant ligand–receptor pairs with a *P* value <0.01.

### Survival analysis

The TCGA OC data and microarray data of GSE9891, GSE19829 to GPL8300 were used to evaluate the prognostic performance of gene sets derived from different EC clusters. We used the mean expression of signature genes for distinct cell clusters to evaluate the enrichment of corresponding EC types in patients diagnosed with HGSOC. Specifically, for *IL13RA1*^+^ and *VCAM1*^+^ EC clusters, we used the top 20 differentially expressed genes of these two clusters as signature genes to define their signatures, (provided in Source Data Fig. 7f). We performed survival analysis using the Cox proportional hazards model implemented in the R package survival (v.3.2.3) to correct patient age and plotted Kaplan–Meier survival curves using the R function ggsurvplot.

### Flow cytometry

**Extended Data Fig. 10:**
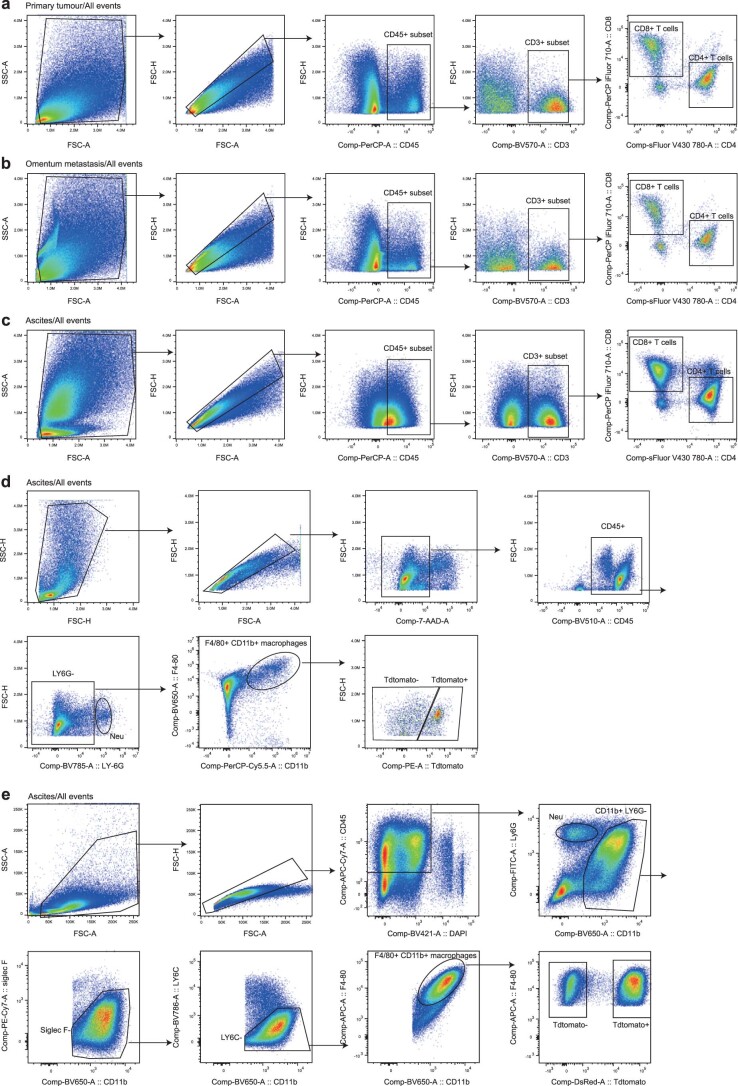
Gating strategies for T cells or macrophages. **a-c**, Gating strategies for T cells in different samples of HGSOC patients, including primary tumor (**a**), omentum metastasis (**b**), and ascites (**c**). **d-e**, Gating strategies for macrophages in ascites of tumor-bearing mice used for proportion analysis of macrophages with different origins (**d**) and sorting out embryonic-origin and monocyte-derived macrophages for bulk RNA-seq (**e**).

Fresh human tumor samples were cut into approximately 1-mm^3^ pieces in RPMI-1640 medium (Invitrogen) with 10% FBS (Gibco) and enzymatically digested with the MACS Tumor Dissociation kit (Miltenyi) for 30 min using a gentleMACS Octo Dissociator (Miltenyi) at 37 °C. Dissociated cells were subsequently passed through a 70-μm cell strainer (BD) and centrifuged at 400*g* for 10 min. The pelleted cells were then resuspended in red blood cell lysis buffer (Miltenyi) and incubated on ice for 5 min to lyse red blood cells. After washing twice with PBS (Invitrogen), cell pellets were resuspended in FACS buffer and kept on ice until staining. Human ascites samples were collected in 50-ml conical tubes (BD) and mouse ascites samples were collected by syringe extraction from terminally anesthetized mice. The samples were centrifuged at 400*g* for 10 min to obtain cell precipitation. The pelleted cells were then lysed using the above-mentioned procedures. After washing twice with PBS (Invitrogen), cell pellets were resuspended in FACS buffer and keep on ice until staining. Antibodies used to analyze T cells included PerCP-conjugated CD45 (1:200 dilution, Invitrogen), BV570-conjugated CD3 (1:100 dilution, BioLegend), SFV430/780-conjugated CD4 (1:100 dilution, Yuanqi), PerCP-iF710-conjugated CD8 (1:100 dilution, Yuanqi), BV480-conjugated CD25 (1:100 dilution, BD), PE-Cy5-conjugated CD127 (1:100 dilution, BioLegend) and SB702-conjugated PD-1 (1:100 dilution, Invitrogen). Antibodies used to gate macrophages in mouse ascites included BV510-conjugated CD45 (1:200 dilution, BD), BV785-conjugated Ly6G (1:200 dilution, BD), PerCP-Cy5.5-conjugated CD11b (1:200 dilution, BD), BV650-conjugated F4/80 (1:200 dilution, BioLegend), BV421-conjugated TIM4 (1:200 dilution, BD), PE-CY7-conjugated CD163 (1:200 dilution, BioLegend) and 7AAD Viability Staining Solution (BD). The tdTomato signal was detected via the PE channel. Cells were maintained at 4 °C and analyzed on Cytek Aurora flow cytometer (Cytek Biosciences). Data were collected in SpectroFlo (v.3.0.0) and analyzed in FlowJo (v.10.6.2). Gating strategies of T cells are shown in Extended Data Fig. 10a–c and gating strategies for macrophages used for proportion analysis are presented in Extended Data Fig. 10d.

### Bulk RNA-seq data generation and analysis

FACS analysis was used to isolate macrophages with or without tdTomato signals from mouse malignant ascites, performed on a BD Aria III instrument. Antibodies used in this section were APC/cyanine7-conjugated CD45 (1:200 dilution, BD), FITC-conjugated Ly6G (1:200 dilution, BioLegend), PE/cyanine7-conjugated Siglec F (1:200 dilution, Invitrogen), BV785-conjugated Ly6C (1:200 dilution, BioLegend), BV650-conjugated CD11b (1:200 dilution, BioLegend), Alexa Fluor 647-conjugated F4/80 (1:200 dilution, BioLegend) and 4,6-diamidino-2-phenylindole (Invitrogen). Expression levels of these molecules were gated by their negative controls of unstained cells and positive controls of cells stained by each antibody. Expression levels of tdTomato were gated by negative controls of wild-type mice without tdTomato signals. Gating strategies are presented in Extended Data Fig. 10e. Based on FACS analysis, macrophages were sorted into 96-well plates (Axygen) chilled to 4 °C, prepared with lysis buffer with 1 μl 10 mM dNTP mix (Invitrogen), 1 μl 10 μM Oligo dT primer, 1.9 μl 1% Triton X-100 (Sigma) and 0.1 μl 40 U μl^−1^ RNase Inhibitor (Takara). The cell lysates were sealed and stored frozen at −80 °C immediately. Transcriptome amplifications were performed according to protocol. The External RNA Controls Consortium spike-in controls (Ambion; 1:4,000,000 dilution) were added into each well before reverse transcription. Amplified cDNA products were purified with Agencourt XP DNA beads (Beckman). Quality control (QC) was performed following the first round of purification, which included the detection of *GAPDH* by qPCR and fragment analysis by Fragment Analyzer (AATI). For single-cell samples of high quality after QC (cycle threshold < 30), the DNA products were further purified with 0.5× Agencourt XP DNA beads and the concentration of each sample was quantified using Qubit HsDNA kits (Invitrogen). Multiplex (384-plex) libraries were constructed and amplified using the TruePrep DNA Library Prep Kit V2 for Illumina (Vazyme Biotech). The libraries were then purified with Agencourt XP DNA beads and pooled for quality assessment by Fragment Analyzer. Purified libraries were then analyzed by an Illumina Hiseq 4000 sequencer with 150-bp paired-end reads. Fastp was used to get the clean reads. Read mapping was performed using STAR (Bulk RNA-seq, v.2.7.2a) using the mouse reference genome (mm10). Gene level quantification was completed using Subread featureCounts (bulk RNA-seq).

### Multicolor immunohistochemistry of human tissues

Human tissue specimens, including tumor samples and ascites cell precipitation, were provided by Xinhua Hospital Affiliated to Shanghai Jiaotong University School of Medicine. The specimens were collected within 30 min after the tumor resection and fixed in formalin for 48 h. Dehydration and embedding in paraffin was performed following routine methods. Paraffin blocks were cut into 5-μm slices and adhered to slide glass. Sections were then placed into a paraffin oven at 70 °C for 1 h before deparaffinization in xylene and successive rehydration in 100%, 90%, 70% alcohol. Antigen was retrieved by citric acid buffer (pH 6.0) in a water bath at 95 °C for 20 min. Endogenous peroxidase was inactivated by incubation in 3% H_2_O_2_ for 15 min. Following preincubation with 10% normal goat serum to block nonspecific sites for 30 min, sections were incubated with primary antibodies in a humidified chamber at 4 °C overnight. The antibodies used in the validation of *DES*^+^ mesothelial cells in ascites cell precipitation were anti-MSLN (1:250 dilution, Cell Signaling Technology), anti-UPK3B (1:20 dilution, Abcam), anti-WT1 (1:100 dilution, Cell Signaling Technology) and anti-DES (1:2,000 dilution, Abcam). The antibodies used in the validation of two macrophage subtypes (M07_Macro-EREG and M10_Macro-C1QA) in ovarian tumor tissue were anti-CD68 (1:400 dilution, Cell Signaling Technology), anti-SPP1 (1:2,000 dilution, Abcam), anti-EREG (1:100 dilution, Lifespan Biosciences), anti-IL1B (1:100 dilution, Cell Signaling Technology), anti-C1QA (1:1,000 dilution, Abcam), anti-RGS2 (1:200 dilution, Abcam) and anti-MARCO (1:200 dilution, Lifespan Biosciences). After the sections were washed with PBS twice for 5 min, the antigenic binding sites were visualized using the PhenoImager Fusion (Akoya) with the Phenochart viewer software (v.1.10) according to the manufacturer’s protocol.

### Statistics and reproducibility

No statistical methods were used to predetermine sample sizes and the experiments were not randomized. The investigators were not blinded to allocation during the experiments and outcome assessments. Data collection and analysis were not performed blinded. No data were excluded from the analyses. Statistical analyses were performed using R (v.3.6.1) and GraphPad Prism (v.9.0). One-sided or two-sided unpaired Student’s *t*-tests, two-sided Wilcoxon tests, two-sided unpaired limma-moderated *t*-tests and Kruskal–Wallis tests were used to evaluate significance, as indicated in figure legends. *P* < 0.05 was considered statistically significant. Data distribution was assumed to be normal, but this was not formally tested.

### Reporting summary

Further information on research design is available in the Nature Portfolio Reporting Summary linked to this article.

## Supplementary information

Reporting Summary

Supplementary Table 1 Characteristics of patients and specimens included in our study.

## Data Availability

The scRNA-seq and scTCR-seq data supporting the findings of this study have been deposited at GSA-Human under accession code PRJCA005422, with the processed data deposited in Mendeley Data (10.17632/rc47y6m9mp.1)^65^. An interactive web portal for analysis and visualization of single-cell data is available at http://ov.cancer-pku.cn/. Bulk-RNA-seq data of mice are available from NCBI Gene Expression Omnibus under accession no. GSE223121. Previously published microarray data analyzed together are available under accession codes GSE9891 and GSE19829 to GPL8300. All other supporting data of this study are available from the corresponding author on reasonable request. Source data are provided with this paper.

## Source data

Source Data Fig. 1 Statistical Source Data.
Source Data Fig. 2 Statistical Source Data.
Source Data Fig. 3 Statistical Source Data.
Source Data Fig. 4 Statistical Source Data.
Source Data Fig. 5 Statistical Source Data.
Source Data Fig. 6 Statistical Source Data.
Source Data Fig. 7 Statistical Source Data.
Source Data Fig. 8 Statistical Source Data.
Source Data Extended Data Fig. 1 Statistical Source Data.
Source Data Extended Data Fig. 3 Statistical Source Data.
Source Data Extended Data Fig. 4 Statistical Source Data.
Source Data Extended Data Fig. 5 Statistical Source Data.
Source Data Extended Data Fig. 7 Statistical Source Data.

## Code availability

No new algorithms were developed for this study. All codes generated for analysis are available.
