# Supplementary material for: Single-cell analyses implicate ascites in remodeling the ecosystems of primary and metastatic tumors in ovarian cancer
Source: Nat Cancer. 2023 Jul 24;4(8):1138–56. doi: 10.1038/s43018-023-00599-8 (PMC10447252; doi:10.1038/s43018-023-00599-8)
Supplement: Supplementary file 1 — Reporting Summary [file 43018_2023_599_MOESM1_ESM.pdf]

nature portfolio

Corresponding author(s): *Xipeng Wang, Zemin Zhang, Ziyi Li*

Last updated by author(s): *Apr 26, 2023*

Reporting Summary

Nature Portfolio wishes to improve the reproducibility of the work that we publish. This form provides structure for consistency and transparency in reporting. For further information on Nature Portfolio policies, see our Editorial Policies and the Editorial Policy Checklist.

Statistics

For all statistical analyses, confirm that the following items are present in the figure legend, table legend, main text, or Methods section.

n/a	Confirmed	
☐	☒	The exact sample size (n) for each experimental group/condition, given as a discrete number and unit of measurement
☐	☒	A statement on whether measurements were taken from distinct samples or whether the same sample was measured repeatedly
☐	☒	The statistical test(s) used AND whether they are one- or two-sided<br>*Only common tests should be described solely by name; describe more complex techniques in the Methods section.*
☒	☐	A description of all covariates tested
☐	☒	A description of any assumptions or corrections, such as tests of normality and adjustment for multiple comparisons
☐	☒	A full description of the statistical parameters including central tendency (e.g. means) or other basic estimates (e.g. regression coefficient) AND variation (e.g. standard deviation) or associated estimates of uncertainty (e.g. confidence intervals)
☐	☒	For null hypothesis testing, the test statistic (e.g. F, t, r) with confidence intervals, effect sizes, degrees of freedom and P value noted<br>*Give P values as exact values whenever suitable.*
☒	☐	For Bayesian analysis, information on the choice of priors and Markov chain Monte Carlo settings
☒	☐	For hierarchical and complex designs, identification of the appropriate level for tests and full reporting of outcomes
☒	☐	Estimates of effect sizes (e.g. Cohen's d, Pearson's r), indicating how they were calculated

Our web collection on statistics for biologists contains articles on many of the points above.

Software and code

Policy information about availability of computer code

Data collection	*Cell Ranger for 10X Genomics (version 3.0.2), SpectroFlo (version 3.0.0) were used for data collection.*
Data analysis	*The following software was used in this study:<br>R (v 3.6.1), GraphPad Prism (v 9.0), python (v3.6.13), Seurat (v3.1.4), Harmony (v1.0), Startrac (v 0.1.0), Scanpy (v1.7.2), cellphoneDB (v3.0.0), rstatix (v 0.7.0), clusterProfiler (v 3.14.3), STAR(v 2.7.2a),miloR (v 1.5.0), inferCNV (v1.2.1), Survival (v 3.2.3), FlowJo (v 10.6.2), and PhenochartTM viewer software (v 1.10).*

For manuscripts utilizing custom algorithms or software that are central to the research but not yet described in published literature, software must be made available to editors and reviewers. We strongly encourage code deposition in a community repository (e.g. GitHub). See the Nature Portfolio guidelines for submitting code & software for further information.

Data

Policy information about availability of data

All manuscripts must include a data availability statement. This statement should provide the following information, where applicable:

- Accession codes, unique identifiers, or web links for publicly available datasets
- A description of any restrictions on data availability
- For clinical datasets or third party data, please ensure that the statement adheres to our policy

ScRNA-seq and scTCR-seq data that support the findings of this study have been deposited in the GSA for Human of China National Center for Bioinformation (CNCB)

March 2021

under accession codes PRJCA005422 , with the processed data deposited in Mendeley Data (DOI:10.17632/rc47y6m9mp.1). An interactive website for analyzing and visualizing the scRNA-seq data is available at http://ov.cancer-pku.cn/. Furthermore, cellphoneDB database v2.0.0 was used for ligand-receptor interaction analysis and V(D)J Reference "GRCh38-alts-ensembl" was used for TCR analysis in our study. Bulk-RNA-seq data of mice are available from NCBI-GEO under the accession number GSE223121. Previously published microarray data analyzed together were available under accession code GSE9891, and GSE19829-GPL8300. Besides, TCGA（https://portal.gdc.cancer.gov/） OV data were also used to analyze the overall survival of HGSOC patients divided by signatures of certain endothelial cells in our study. Other data supporting the findings in this study are available from the corresponding authors on reasonable request.

Human research participants

Policy information about studies involving human research participants and Sex and Gender in Research.

Reporting on sex and gender	*The findings of our study about ovarian cancer only apply to females.*
Population characteristics	*Fourteen female patients pathologically diagnosed with ovarian cancer including ten high grade serous ovarian cancer (HGSOC; patients HGSOC1-10), one endometrioid carcinoma of the ovary (ECO; patient ECO1), one ovarian clear cell carcinoma (OCCC; patient OCCC1), one undifferentiated ovarian carcinoma (UOC; patient UOC1), and one ovarian carcinosarcoma (patient C1) were enrolled in this study. The patients HGSOC3, HGSOC6, HGSOC7 and ECO1 were platinum resistant (non-responsive), whereas the other patients but one patient diagnosed as UOC1 were platinum sensitive (responsive). Patients ranged in age from 43 to 82 years old, with a median age of 62 years. Five more patients pathologically diagnosed with HGSOC (patients HGSOC11-15) were enrolled in this study for flow cytometry analyses of T cells. The available clinical metadata of these patients are summarized in Supplementary Table 1.*
Recruitment	*Ovarian cancer patients in Xinhua Hospital Affiliated to Shanghai Jiaotong University School of Medicine and Fudan University Shanghai Cancer Center preparing for surgery were consented to collect bio-specimens during surgery. Patients who were diagnosed with ovarian cancer and agreed to sign the consent file were recruited in our study without any self-selection.*
Ethics oversight	*This study complies with all relevant ethical regulations and was approved by the Ethics Committee of Xinhua Hospital Affiliated to Shanghai Jiaotong University School of Medicine and Fudan University Shanghai Cancer Center. Written informed consent was provided by all participants.*

Note that full information on the approval of the study protocol must also be provided in the manuscript.

Field-specific reporting

Please select the one below that is the best fit for your research. If you are not sure, read the appropriate sections before making your selection.

☒ Life sciences ☐ Behavioural & social sciences ☐ Ecological, evolutionary & environmental sciences

For a reference copy of the document with all sections, see nature.com/documents/nr-reporting-summary-flat.pdf

Life sciences study design

All studies must disclose on these points even when the disclosure is negative.

Sample size	*No statistical methods were used to pre-determine the sample size. Sample size were determined based on published papers and previous experience, and described in the figure legends or Methods. Since samples used for scRNA-seq were human subjects, we collected as many samples as possible within our timeframe. For other studies in our project, a sample size of n > 3 would allow for adequate analysis to reach meaningful conclusions of data.*
Data exclusions	*No data was excluded from the analyses.*
Replication	*The single-cell sequencing for each tumour sample was performed in one experimental run since the cell counts from a given sample are usually low, and the cells cannot be analyzed more than once. Totally n=13 primary tumour samples, n=5 metastatic tumour samples, n=10 ascites samples, n=6 blood samples and n=5 lymph node samples were collected for scRNA-seq successfully. The flow cytometry experiments for human T cells were performed in one experimental run for each patient. But there were more than one patient (n=5) enrolled in this study to verify the reproducibility and all experiments were done successfully. The flow cytometry experiments of mouse ascites were also performed in one experimental run for the limited number of cells captured from ascites in one mouse. But there were more than one mouse ascites sample (n=4) analyzed successfully in this study to verify the reproducibility. And bulk RNA-seq analysis of ascites-derived macrophages using mouse models were successfully performed in 4 independent experimental runs.The IHC experiments for each tumour/ascites cell pellet sample were performed in one experimental run successfully,with n=3 tumour samples to verify the reproducibility.*
Randomization	*This study relied on the use of human biospecimens with no interventions performed on the human subjects. Randomization was not applicable. For in vivo mouse experiments, randomization was not required because all mice belonged to one experimental group and were seeded with tumour cells equally.*
Blinding	*The diagnosis of tumour was known when we obtaining the biosamples. There is no intervention to patients recruited, and it's not a clinical trail. Blinding is not applicable. For remaining experiments in our study, no blinding was used.*

Behavioural & social sciences study design

All studies must disclose on these points even when the disclosure is negative.

Study description	*Not applicable.*
Research sample	*Not applicable.*
Sampling strategy	*Not applicable.*
Data collection	*Not applicable.*
Timing	*Not applicable.*
Data exclusions	*Not applicable.*
Non-participation	*Not applicable.*
Randomization	*Not applicable.*

Ecological, evolutionary & environmental sciences study design

All studies must disclose on these points even when the disclosure is negative.

Study description	*Not applicable.*
Research sample	*Not applicable.*
Sampling strategy	*Not applicable.*
Data collection	*Not applicable.*
Timing and spatial scale	*Not applicable.*
Data exclusions	*Not applicable.*
Reproducibility	*Not applicable.*
Randomization	*Not applicable.*
Blinding	*Not applicable.*

Did the study involve field work? ☐ Yes ☒ No

Field work, collection and transport

Field conditions	*Not applicable.*
Location	*Not applicable.*
Access & import/export	*Not applicable.*
Disturbance	*Not applicable.*

Reporting for specific materials, systems and methods

We require information from authors about some types of materials, experimental systems and methods used in many studies. Here, indicate whether each material, system or method listed is relevant to your study. If you are not sure if a list item applies to your research, read the appropriate section before selecting a response.

Materials & experimental systems

n/a	Involved in the study
☐	☒ Antibodies
☐	☒ Eukaryotic cell lines
☒	☐ Palaeontology and archaeology
☐	☒ Animals and other organisms
☒	☐ Clinical data
☒	☐ Dual use research of concern

Methods

n/a	Involved in the study
☒	☐ ChIP-seq
☐	☒ Flow cytometry
☒	☐ MRI-based neuroimaging

Antibodies

Antibodies used

Antibody (Flow cytometry)
For human samples
CD45 PerCP (Cat# MHCD4531, Lot# 2109773, Clone HI30, Invitrogen); dilution 1:200
CD3 BV570 (Cat# 300436, Lot# B334945, Clone UCHT1, Biolegend); dilution 1:100
CD4 SFV430/780 (Cat# 20210513-16, Lot# 2740344, Clone SK3, Yuanqi); dilution 1:100
CD8 PerCP-iF710 (Cat# 210823-12, Lot# 2750931, Clone SK1, Yuanqi); dilution 1:100
CD25 BV480 (Cat# 566102, Lot# 1195023, Clone M-A251, BD Biosciences); dilution 1:100
CD127 PE-Cy5 (Cat# 351324, Lot# B359489, Clone A019D5, Biolegend); dilution 1:100
PD-1 SB702 (Cat# 67-2799-42, Lot# 2378852, Clone J105, Invitrogen); dilution 1:100
For mouse samples
CD45 BV510 (Cat# 563891, Lot# 9344071, Clone 30-F11, BD Biosciences); dilution 1:200
Ly-6G BV786 (Cat# 740953, Lot# 1348402, Clone 1A8, BD Biosciences); dilution 1:200
CD11b PerCP-Cy5.5 (Cat# 550993, Lot# 1033685, Clone M1/70, BD Biosciences); dilution 1:200
F4/80 BV650 (Cat# 123149, Lot# B345158, Clone BM8, BioLegend); dilution 1:200
TIM4 BV421 (Cat#742773, Lot# 1319758, Clone 21H12, BD Biosciences); dilution 1:200
CD163 PE-CY7 (Cat#155319, Lot# B349540, Clone S15049, BioLegend); dilution 1:200

Antibody (FACS) for mouse samples
CD45 APC/Cyanine7 (Cat# 557659, Lot# 396774, Clone 30-F11, BD Biosciences); dilution 1:200
Ly6G FITC (Cat# 127606, Lot# 1236494, Clone 1A8, BioLegend); dilution 1:200
Siglec F PE/Cyanine7 (Cat# 25-1702-82, Lot# 2802251, Clone 1RNM44N, Invitrogen); dilution 1:200
Ly6C BV785 (Cat# 128041, Lot# 2565852, Clone HK1.4, BioLegend); dilution 1:200
CD11b BV650 (Cat# 101259, Lot# 2566568, Clone M1/70, BioLegend); dilution 1:200
F4/80 Alexa Fluor 647 (Cat# 123122, Lot# 893480, Clone BM8, BioLegend); dilution 1:200

Antibody (multicolour IHC)
Rabbit anti-MSLN (Cat# 99966T, Lot# 1, Clone D9R5G, Cell Signaling Technology); dilution 1:250
Rabbit anti-UPK3B (Cat# ab197368, Lot# GR282628-9, Polyclonal, Abcam); dilution 1:20
Rabbit anti-WT1 (Cat# 83535T, Lot# 1, Clone D8I7F, Cell Signaling Technology); dilution 1:100
Rabbit anti-DES (Cat# ab32362, Lot# GR152193-50, Clone Y66, Abcam); dilution 1:2000
Rabbit anti-CD68 (Cat# 76437T, Lot# 4, Clone D4B9C, Cell Signaling Technology); dilution 1:400
Rabbit anti-SPP1 (Cat# ab283656, Lot# GR3400643-6, Clone RM1018, Abcam); dilution 1:2000
Rabbit anti-EREG (Cat# LS-C778687, Lot# 210021, Polyclonal, Lifespan Biosciences); dilution 1:100
Mouse anti-IL1B (Cat# 12242S, Lot# 1, Clone 3A6, Cell Signaling Technology); dilution 1:100
Rabbit anti-C1QA (Cat# ab189922, Lot# GR173398-1, Clone EPR14634, Abcam); dilution 1:1000
Rabbit anti-RGS2 (Cat# ab155762, Lot# GR135813-20, PolyClonal, Abcam); dilution 1:200
Rabbit anti-MARCO (Cat# LS-C676024, Lot# 186303, Polyclonal, Lifespan Biosciences); dilution 1:200

Validation

All antibodies were commercially available and validated by manufacturer. Based on the information from the manufacturer's website,the validation information for species and application is listed below. And all antibodies were validated based on the manufacturer's instructions using mouse ascites (for FACS antibodies)/human ovarian tumour samples(for multicolour IHC antibodies).
Antibodies (Flow cytometry)
For human samples
CD45 PerCP (Cat# MHCD4531, Invitrogen) https://www.thermofisher.cn/cn/zh/antibody/product/CD45-Antibody-clone-HI30-Monoclonal/MHCD4531
CD3 BV570 (Cat# 300436, Biolegend) https://www.biolegend.com/en-us/products/brilliant-violet-570-anti-human-cd3-antibody-7368
CD4 SFV430/780 (Cat# 20210513-16, Yuanqi)
CD8 PerCP-iF710 (Cat# 210823-12, Yuanqi)
CD25 BV480 (Cat# 566102, BD Biosciences) https://www.bdbiosciences.com/en-us/search-results?searchKey=566102
CD127 PE-Cy5 (Cat# 351324, Biolegend) https://www.biolegend.com/en-us/products/pe-cyanine5-anti-human-cd127-il-7ralpha-antibody-7504
PD-1 SB702 (Cat# 67-2799-42, Invitrogen) https://www.thermofisher.cn/cn/zh/antibody/product/CD56-NCAM-Antibody-clone-TULY56-Monoclonal/67-0566-42
For mouse samples
CD45 BV510 (Cat# 563891, BD Biosciences) https://www.bdbiosciences.com/zh-cn/search-results?searchKey=563891
Ly-6G BV786 (Cat# 740953, BD Biosciences) https://www.bdbiosciences.com/zh-cn/search-results?searchKey=740953
CD11b PerCP-Cy5.5 (Cat# 550993, BD Biosciences) https://www.bdbiosciences.com/zh-cn/search-results?searchKey=550993
F4/80 BV650 (Cat# 123149, BioLegend) https://www.biolegend.com/en-us/products/brilliant-violet-650-anti-mouse-f4-80-antibody-10630

TIM4 BV421 (no. BD742773) https://www.bdbiosciences.com/en-us/products/reagents/flow-cytometry-reagents/research-reagents/single-color-antibodies-ruo/bv421-rat-anti-mouse-tim-4.742773
CD163 PE-CY7 (no. BL155319) https://www.biolegend.com/en-us/products/pe-cyanine7-anti-mouse-cd163-antibody-20615

Antibody (FACS) for mouse samples
CD45 APC/Cyanine7 (Cat# 557659, BD Biosciences) https://www.bdbiosciences.com/zh-cn/products/reagents/flow-cytometry-reagents/research-reagents/single-color-antibodies-ruo/apc-cy-7-rat-anti-mouse-cd45.557659
Ly6G FITC (Cat# 127606, BioLegend) https://www.biolegend.com/en-us/products/fitc-anti-mouse-ly-6g-antibody-4775
Siglec F PE/Cyanine7 (Cat# 25-1702-82, Invitrogen) https://www.thermofisher.cn/cn/zh/antibody/product/CD170-Siglec-F-Antibody-clone-1RNM44N-Monoclonal/25-1702-82
Ly6C BV785 (Cat# 128041, BioLegend) https://www.biolegend.com/en-us/products/brilliant-violet-785-anti-mouse-ly-6c-antibody-11982
CD11b BV650 (Cat# 101259, BioLegend) https://www.biolegend.com/en-us/products/brilliant-violet-650-anti-mouse-human-cd11b-antibody-7638
F4/80 Alexa Fluor 647 (Cat# 123122, BioLegend) https://www.biolegend.com/en-us/products/alexa-fluor-647-anti-mouse-f4-80-antibody-4074

Antibodise for multicolour IHC
Rabbit anti-MSLN (1:250, Cat# 99966T, Cell Signaling Technology) React with: Human; Suitable for: WB, IHC
Rabbit anti-UPK3B (1:20, Cat# ab197368, Abcam) React with: Human; Suitable for: WB, IHC-P
Rabbit anti-WT1 (1:100, Cat# 83535T, Cell Signaling Technology) React with: Human; Suitable for: WB, IP, IHC, IF
Rabbit anti-DES (1:2000, Cat# ab32362, Abcam) React with: Mouse, Rat, Guinea pig, Human; Suitable for: WB, IHC-P, ICC
Rabbit anti-CD68 (1:400, Cat# 76437T, Cell Signaling Technology) React with: Human; Suitable for: IHC, IF, F
Rabbit anti-SPP1 (1:2000, Cat# ab283656, Abcam) React with: Mouse, Human, Recombinant fragment; Suitable for: IHC-P, WB, IP
Rabbit anti-EREG (1:100, Cat# LS-C778687, Lifespan Biosciences) React with: Mouse, Human, Rat; Suitable for: IHC-P, WB, IP
Mouse anti-IL1B (1:100, Cat# 12242S, Cell Signaling Technology) React with: Human, Mouse; Suitable for: WB, IHC
Rabbit anti-C1QA (1:1000, Cat# ab189922, Abcam) React with: Rat, Human; Suitable for: IHC-P, ELISA
Rabbit anti-RGS2 (1:200, Cat# ab155762, Abcam) React with: Mouse, Rat, Human; Suitable for: IHC-P, WB, ICC/IF
Rabbit anti-MARCO (1:200, Cat# LS-C676024, Lifespan Biosciences) React with: Human; Suitable for: IHC, IHC-P, WB, ELISA

Eukaryotic cell lines

Policy information about cell lines and Sex and Gender in Research

Cell line source(s)	ID8 is a mouse ovarian surface epithelial cell line usually used to establish the mouse model for human ovarian cancer. The cell line used in the study was purchased from FuHeng Biology,Shanghai,China.
Authentication	The cell lines used in the study was authenticated with STR profiling.
Mycoplasma contamination	The cell line used in the study was confirmed to be mycoplasma nagative.
Commonly misidentified lines (See ICLAC register)	No any commonly misidentified cell lines were used in the study.

Palaeontology and Archaeology

Specimen provenance	Not applicable.
Specimen deposition	Not applicable.
Dating methods	Not applicable.

☐ Tick this box to confirm that the raw and calibrated dates are available in the paper or in Supplementary Information.

Ethics oversight	Not applicable.

Note that full information on the approval of the study protocol must also be provided in the manuscript.

Animals and other research organisms

Policy information about studies involving animals; ARRIVE guidelines recommended for reporting animal research, and Sex and Gender in Research

Laboratory animals	Strains used in this study: C57BL/6(Ms4a3 and Rosatomato transgenic monocyte fate-mapping mouse), female, mouse, 4-5weeks old.
Wild animals	We have not used any wild animals in this study.
Reporting on sex	The findings of our study about ovarian cancer only apply to females. The mice used in our experiments were all females judged by the distance between the external genitalia and the anus which is shorter in females than in males at all postnatal stages.

Field-collected samples | *No field-collected samples used.*

Ethics oversight | *All the procedures were approved by the Institutional Animal Care and Use Committee (IACUC) of Model Animal Research Center, Xinhua Hospital, Shanghai Jiaotong University School of Medicine and were performed in compliance with the guidelines for the care and use of laboratory animals. The maximal tumour burden was not exceeded for mouse tumour experiments on the requirement of our ethics committee.*

Note that full information on the approval of the study protocol must also be provided in the manuscript.

Clinical data

Policy information about clinical studies

All manuscripts should comply with the ICMJE guidelines for publication of clinical research and a completed CONSORT checklist must be included with all submissions.

Clinical trial registration | *Not applicable.*

Study protocol | *Not applicable.*

Data collection | *Not applicable.*

Outcomes | *Not applicable.*

Dual use research of concern

Policy information about dual use research of concern

Hazards

Could the accidental, deliberate or reckless misuse of agents or technologies generated in the work, or the application of information presented in the manuscript, pose a threat to:

No	Yes	
☒	☐	Public health
☒	☐	National security
☒	☐	Crops and/or livestock
☒	☐	Ecosystems
☒	☐	Any other significant area

Experiments of concern

Does the work involve any of these experiments of concern:

No	Yes	
☒	☐	Demonstrate how to render a vaccine ineffective
☒	☐	Confer resistance to therapeutically useful antibiotics or antiviral agents
☒	☐	Enhance the virulence of a pathogen or render a nonpathogen virulent
☒	☐	Increase transmissibility of a pathogen
☒	☐	Alter the host range of a pathogen
☒	☐	Enable evasion of diagnostic/detection modalities
☒	☐	Enable the weaponization of a biological agent or toxin
☒	☐	Any other potentially harmful combination of experiments and agents

ChIP-seq

Data deposition

☐ Confirm that both raw and final processed data have been deposited in a public database such as GEO.

☐ Confirm that you have deposited or provided access to graph files (e.g. BED files) for the called peaks.

Data access links
May remain private before publication. | *Not applicable.*

Files in database submission | *Not applicable.*

Genome browser session
(e.g. UCSC) | Not applicable.

Methodology

Replicates: Not applicable.

Sequencing depth: Not applicable.

Antibodies: Not applicable.

Peak calling parameters: Not applicable.

Data quality: Not applicable.

Software: Not applicable.

Flow Cytometry

Plots

Confirm that:

☒ The axis labels state the marker and fluorochrome used (e.g. CD4-FITC).

☒ The axis scales are clearly visible. Include numbers along axes only for bottom left plot of group (a 'group' is an analysis of identical markers).

☒ All plots are contour plots with outliers or pseudocolor plots.

☒ A numerical value for number of cells or percentage (with statistics) is provided.

Methodology

Sample preparation: *Standard flow cytometry protocal was used in this study. Human samples were collected at the time of operation. Fresh human tumour samples were cut into pieces and enzymatically digested with the MACS Tumour Dissociation Kit (Miltenyi). The pelleted cells after centrifugation were then resuspended in red blood cell lysis buffer (Miltenyi) to lyse red blood cells. Human ascites samples were collected in 50-ml conical tubes (BD). Mouse ascites were collected by syringe extraction from terminally anaesthetized mice. Red blood cell lysis buffer (Miltenyi) was used to remove the interference of red cells in ascites samples. After washing with PBS (Invitrogen), the cell pellets were resuspended in FACs buffer, keep on ice until staining. Detailed information is available in the Methods section.*

Instrument: *Cytek Aurora flow cytometer (Cytek Biosciences) and BD Aria III.*

Software: *SpectroFlo (version 3.0.0) used for data collection. FlowJo (version10.6.2) used for data analysis.*

Cell population abundance: *Around 20,000-100,000 cells in each experiment was achieved. Purity was determined by flow cytometer during and after the sort.*

Gating strategy: *To analyze the proportion of certain T subtypes in tumours and ascites of HGSOC patients, cells were gated as follows:*
1. First gated based on physical parameters SSC-A/FSC-A.
2. FSC-H/FSC-A to identify single cells from doublets/complexes.
3. CD45+ for general immune cells.
4. CD3+ for T cells.
5. CD4+ CD8- for CD4+ T cells; CD8+ CD4- for CD8+ T cells.

To validate the proportion of embronic-origin macrophages in ovarian tumour ascites using monocyte fate-mapping mouse, cells were gated as follows:
1. First gated based on physical parameters SSC-H/FSC-H.
2. FSC-H/FSC-A to identify single cells from doublets/complexes.
3. 7-ADD for live cells.
4. CD45+ for general immune cells.
5. Ly6G- to remove interference from neutrophils.
6. F4/80+ and CD11b+ for macrophages.
7. TdTomato+ for monocytes derived macrophages and TdTomato- represents macrophages of embrynic origin.

To sort out embronic-origin and monocyte-derived macrophages in ovarian tumour ascites using monocyte fate-mapping mouse, cells were gated as follows:
1. First gated based on physical parameters SSC-A/FSC-A.
2. FSC-H/FSC-A to identify single cells from doublets/complexes.
3. CD45+ DAPI- for live immune cells.
4. Ly6G- to remove interference from neutrophils.
5. SiglecF- to remove interference from eosinophils.

6. Ly-6C-, F4/80+ and CD11b+ for macrophages.
7. TdTomato+ for monocytes derived macrophages and TdTomato- represents macrophages of embryonic origin.

☒ Tick this box to confirm that a figure exemplifying the gating strategy is provided in the Supplementary Information.

Magnetic resonance imaging

Experimental design

Design type	*Not applicable.*
Design specifications	*Not applicable.*
Behavioral performance measures	*Not applicable.*

Acquisition

Imaging type(s)	*Not applicable.*
Field strength	*Not applicable.*
Sequence & imaging parameters	*Not applicable.*
Area of acquisition	*Not applicable.*

Diffusion MRI ☐ Used ☒ Not used

Preprocessing

Preprocessing software	*Not applicable.*
Normalization	*Not applicable.*
Normalization template	*Not applicable.*
Noise and artifact removal	*Not applicable.*
Volume censoring	*Not applicable.*

Statistical modeling & inference

Model type and settings	*Not applicable.*
Effect(s) tested	*Not applicable.*

Specify type of analysis: ☐ Whole brain ☐ ROI-based ☐ Both

Statistic type for inference (See Eklund et al. 2016)	*Not applicable.*
Correction	*Not applicable.*

Models & analysis

n/a	Involved in the study
☒	☐ Functional and/or effective connectivity
☒	☐ Graph analysis
☒	☐ Multivariate modeling or predictive analysis

Functional and/or effective connectivity	*Not applicable.*
Graph analysis	*Not applicable.*
Multivariate modeling and predictive analysis	*Not applicable.*
